# Gut microbiome of mealworms (*Tenebrio molitor* Larvae) show similar responses to polystyrene and corn straw diets

**DOI:** 10.1186/s40168-023-01550-w

**Published:** 2023-05-05

**Authors:** Tursunay Mamtimin, Huawen Han, Aman Khan, Pengya Feng, Qing Zhang, Xiaobiao Ma, Yitian Fang, Pu Liu, Saurabh Kulshrestha, Toshiro Shigaki, Xiangkai Li

**Affiliations:** 1 Ministry of Education Key Laboratory of Cell Activities and Stress Adaptations, School of Life Science, Lanzhou University, Lanzhou, China; 2 State Key Laboratory of Grassland Agro-Ecosystems, Center for Grassland Microbiome, Lanzhou University, Lanzhou, China; 3 State Key Laboratory of Microbial Metabolism, School of Life Sciences and Biotechnology, Shanghai Jiao Tong University, Shanghai, China; 4 Faculty of Applied Sciences and Biotechnology, Shoolini University, Solan, India; 5 Graduate School of Agricultural and Life Sciences, The University of Tokyo, Tokyo, Japan

**Keywords:** *Tenebrio molitor*, Polystyrene, Corn straw, Microbial community, Metabolic pathway, Biodegradation

## Abstract

**Background:**

Some insects can degrade both natural and synthetic plastic polymers, their host and gut microbes play crucial roles in this process. However, there is still a scientific gap in understanding how the insect adapted to the polystyrene (PS) diet from natural feed. In this study, we analyzed diet consumption, gut microbiota responses, and metabolic pathways of *Tenebrio molitor* larvae exposed to PS and corn straw (CS).

**Results:**

*T. molitor* larvae were incubated under controlled conditions (25 ± 1 °C, 75 ± 5% humidity) for 30 days by using PS foam with weight-, number-, and size-average molecular weight (Mw, Mn, and Mz) of 120.0, 73.2, and 150.7 kDa as a diet, respectively. The larvae exhibited lower PS consumption (32.5%) than CS (52.0%), and these diets had no adverse effects on their survival. The gut microbiota structures, metabolic pathways, and enzymatic profiles of PS- and CS-fed larvae showed similar responses. The gut microbiota of larvae analysis indicated *Serratia* sp., *Staphylococcus* sp., and *Rhodococcus* sp. were associated with both PS and CS diets. Metatranscriptomic analysis revealed that xenobiotics, aromatic compounds, and fatty acid degradation pathways were enriched in PS- and CS-fed groups; laccase-like multicopper oxidases, cytochrome P450, monooxygenase, superoxidase, and dehydrogenase were involved in lignin and PS degradation. Furthermore, the upregulated gene *lac640* in both PS- and CS-fed groups was overexpressed in *E. coli* and exhibited PS and lignin degradation ability.

**Conclusions:**

The high similarity of gut microbiomes adapted to biodegradation of PS and CS indicated the plastics-degrading ability of the *T. molitor* larvae originated through an ancient mechanism that degrades the natural lignocellulose.

Video Abstract

**Graphical Abstract:**

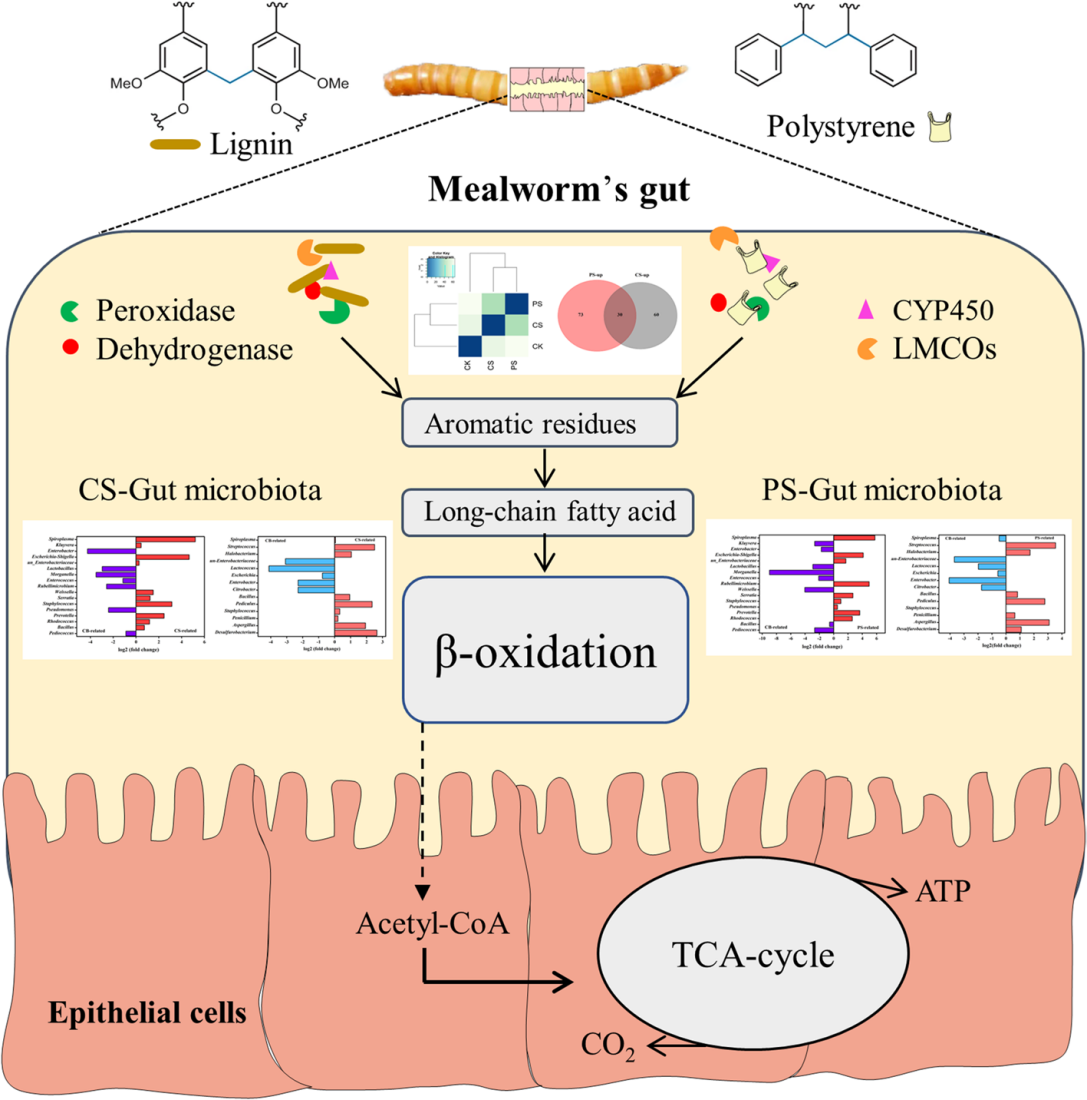

**Supplementary Information:**

The online version contains supplementary material available at 10.1186/s40168-023-01550-w.

## Background

Plastic contamination is continuously increasing due to rapid urbanization and industrialization, and produced about 368 million tons of plastic as reported in 2019 [1]. The widespread usage, low recycling rates, and non-degradable nature of plastics have resulted in their accumulation in the environment [2]. Although mechanical, chemical and physical techniques have been applied to the management of plastic waste, these methods are ineffective and can cause pollution through the emission of different gases and additive chemicals [3]. Therefore, biological technologies have been developed as an eco-friendly alternative for eliminating plastic waste. Several bacteria and fungi are capable of degrading plastic materials at low rates [4, 5]. Recently, numerous studies highlighted the role of some insects and their gut microbes in plastic degradation [6–8]. For example, yellow worms (*Tenebrio molitor*) [9], wax moths (*Galleria mellonella*) [10], and super worms (*Zophobas atratus*) [11] can degrade and metabolize polystyrene (PS), polyethylene (PE), and polyvinyl chloride (PVC) [12]. Other soil invertebrates, land snails (*Achatina fulica)* are capable of ingesting and degrading PS plastics [13], and gut bacteria isolated from earthworms (Lumbricus terrestris) can decay LDPE microplastics [14].

Insect’s diet selection is a long process of adaptation, most insect diets are plants with lignocellulose as the main component [15]. Lignocellulose-feeding insects including termites, locusts and beetles have a natural microbial community in their gut acting as a lignin degrader [16, 17]. As a model insect for screening lignocellulose-degrading microorganisms, many pure cultured microbes, such as *Bacteroides*, *Staphylococcus*, *Streptococcus*, and *Bacillus*, have been isolated from termites [18, 19]. Carbohydrate-active enzymes (CAZymes) were reported to be produced by gut bacteria for lignocellulose degradation, thereby contributing to the growth of insects [20]. The diet of great wax moths (*G. mellonella*) and mealworms (*T. molitor*) typically contains wax and wheat bran, respectively (*Biology: Mealworm-HandWiki*) [21, 22], whereas mealworms can digest lignocellulose-rich crops, including rice straw, rice husk, and corn straw (CS) [23]. Intriguingly, these insects are also capable of ingesting and degrading synthetic plastic, and several plastic-degrading bacteria, such as *Enterobacter asburiae*, *Exiguobacterium* sp., and *Bacillus* sp., have been identified within their guts [7, 10]. The degradation of natural polymers by insects is a natural mechanism that has a long evolutionary history [24]; however, plastics were invented a century ago; this raises questions about how synthetic plastics are metabolized by insects.

Most plastics are derived from crude oil, and the chemical bonds connecting the monomers resemble those found in natural polymers [25, 26]. The fungi *Trametes versicolor* and *Phanerochaete chrysosporium* have been reported can degrade lignin by several extracellular enzymes such as lignin peroxidase (Lip), manganese peroxidase (Mn), and laccase (Lac) [27, 28]. Synthetic plastic PE, polypropylene (PP), and PS share similar chemical structures with lignin, such as aromatic rings and carbon skeletons [26]. These similarities make it possible for lignin-modifying enzymes to degrade these plastics [25, 29]. Some ligninolytic enzymes were produced by *Bacillus* sp. PE3 in the PE containing media [30]. Another study also found that the PS degrading enzyme, hydroquinone peroxidase, was secreted by lignin-degrading bacterium *Azotobacter beijerinckii* HM121 [31]. Moreover, the well-known lignocellulose-degrading fungi *Aspergillus flavus* were isolated from wax moth, and two laccase-like multicopper oxidases (LMCOs) were upregulated under plastic stress [32]. Thus, insect gut microbes may use these natural polymer-degrading mechanisms to break down synthetic plastic polymers as well.

Synthetic plastics and natural lignocellulosic polymers are resistant to the degradation process because of their long chain of inert C–C bonds [33]. The insect *T. molitor* larvae can digest synthetic plastics and natural lignocellulose polymers, their host enzymes and gut microbes play important roles in this process. Thus, we hypothesized that ① the gut microbe responses and metabolic pathways of PS and CS degradation in larvae are similar against the natural polymer CS and the synthetic polymer PS; ② some enzymes in the larval host and gut can degrade both PS and CS; and ③the ability of mealworms to degrade plastics is attributed to their capacity to degrade lignin. In this study, the larvae were fed with PS and CS diets to evaluate their influences on survival and gut microbiota, and cabbage (CB) was used as a control due to the less lignocellulose component rather than wheat bran. The degradation intermediates of PS/CS were detected and characterized via gas chromatography–mass spectrometry (GC − MS) and metabolomics analysis. The metabolic pathways and potential functional enzymes associated with PS and CS degradation were also analyzed by comparative metatranscriptomic sequencing. Furthermore, the differentially expressed genes (DEGs) related to PS and CS degradation were verified by real-time polymerase chain reaction (RT-PCR), and the highly expressed gene *lac640* in both PS- and CS-fed group was overexpressed in *E. coli* to determine its PS and CS degradation ability.

## Methods

### Test materials and mealworm sources

Three different feedstocks, namely, cabbage (CB), PS foam, and CS, were used as a diet for mealworms. The PS foam was purchased from local suppliers (frozen sample packs). According to gel permeation chromatography (GPC) analysis [6], the weight- and number-average molecular weight of the PS was 120,000 Da and 73,178 Da (Additional file 1: S1, Table S1). CS was obtained from corn-cultivating farmlands in Yuzhong County (Lanzhou, China), and CB was purchased from a supermarket as a control. The main characteristics of the CB and CS were shown in Table S1. *T. molitor* larvae were obtained from Insect Breeding Plant (Lanzhou, China). The average weight and length of the larvae were 75–90 mg and 2 cm, respectively.

### Mealworm survival and PS/CS consumption

Primary tests were performed to observe the PS and CS consumption by larvae and their effects on larval survival accordingly [7]. *T. molitor* larvae (*n* = 200) were incubated in a rectangular food-grade polypropylene container (L × W × D: 13 × 7 × 5 cm) under controlled conditions [25 ± 1 °C, 75 ± 5% humidity, and 16:8 (light/dark) photoperiod] with PS (2.0 g) or CS (2.0 g) as the sole diet. Unfed and CB-fed larvae were used as a control (Additional file 1: S1, Fig. S1). To minimize the effects of the previous diet, the larvae were fed CB for at least 3 days, then removed into new containers and kept starved for 48 h [9]. The larval survival rates (SRs), pupation rates (PRs), and the mass loss of all diets were measured for 30 days with an interval of 5 days. Dead larvae and molts were removed from the containers at each measurement point to prevent the dead worms from being eaten by the remaining larvae. Furthermore, the mass balances of PS and CS degradation were calculated accordingly [23, 34], details were shown in the Additional file 1: (S1, M1).

The effects of antibiotic on the PS, CS and CB consumption of larvae were tested using a combination of gentamicin, vancomycin, and ampicillin accordingly [8, 35]. To eliminate the gut microbes of larvae, the above antibiotics were supplemented with the diet at a ratio of 5:3:3 (55 mg/g of bran food) fed to the larvae (*n* = 300 per group, *n* = 3) for 8 days, whereas a control group was fed with bran without antibiotics. The ten mealworms were randomly selected and disinfected with 75% ethanol for 1 min, then washed with sterile water. The gut samples were drawn out and put into the 2 mL tube with 1 mL PBS. After shaking in the vortex for 10 min, the bacterial suspension was diluted to 10^–1^, 10^–2^, 10^–3^ with PBS, coated on the plate, and cultured in the tryptic soy agar (TSA) medium for 24 h. The number of active gut bacteria colonies was counted at 0, 2, 4, 6, and 8 days, respectively [8]. For the uncultured bacteria, normal polymerase chain reaction (PCR, 27F: AGAGTTTGATCCTGGCTCAG, 1492R: GGTTACCTTGTTACGACTT) and real-time quantitative PCR (RT-qPCR, F: AGAGTTTGATCCTGGCTCAG; R: CTGCTGCCTCCCGTAGGAGT) were determined, respectively. Subsequently, the remaining antibiotic-treated mealworms were divided into three groups for feeding on PS, CS and CB, whereas the untreated larvae were also fed the same feedstocks as the control. The SRs and consumption rates (CRs) of with or without antibiotics larvae fed with PS, CS and CB were measured after 5 days, all tests were performed in duplicate.

### Chemical characterization of the frass of PS-/CS-fed larvae and comparative metabolomic analysis

At the end of the 30-day test, frass samples of larvae from each group were collected and stored at − 80 °C for analysis. Fourier transform infrared spectroscopy (FTIR) (Thermo Nicolet NEXUS 670 FTIR, USA) was performed to characterize major functional groups of PS/CS feedstocks (control) and frass from the PS-, CS-fed and unfed larvae in the range of 4000 − 500 cm^−1^. The samples were prepared accordingly [12]. The characterization of thermal variation between the control PS/CS samples and frass from the PS- and CS-fed larvae were performed by thermogravimetric analysis (TGA) using a TG analyzer (Linseis TGA PT1600, Germany). All samples (each group: 5 mg) for TGA were tested from room temperature to 600 °C at a heating rate of 10 °C/min, under a nitrogen atmosphere (> 99.9%) with a flow rate of 10 mL/min [36]. GPC (Waters 1515, U.S.A.) was used to characterize depolymerized polymer molecular weight (number- [Mn], weight- [Mw] and Size- [Mz] averaged molecular weight) in the frass according to the previous methods [36], details were shown in the Additional file 1: (S1, M2). The lignocellulose component of CB, CS feedstocks, and frass of CS-,CB-fed larvae was measured by using Van Soest methods (ANKOM220 Fiber analyzer) [37], as shown in Additional file 1: (S1, M3). Scanning electron microscopy (SEM) and SEM-energy dispersive spectrometry (SEM − EDS, Apreo S, ThermoFisher, U.S.A) were used for observing micro-morphological images of raw CS, PS feedstocks, and frass of PS-, CS-fed larvae.

GC − MS (ThermoFisher, San Jose, California, U.S.A.) was applied to detect the intermediates of CS and PS metabolism. All samples, including control PS, CS and CB feedstocks, intestinal and frass samples of PS-, CS-, CB-fed and unfed larvae, were pretreated with a slightly modified method as previously described [10, 38], details were presented in the Additional file 1: (S1, M4). To further identify the metabolites and metabolic pathways of PS and CS degradation in the larvae, comparative metabolomic analysis was conducted by liquid chromatography − tandem mass spectrometry (LC − MS/MS), as shown in Additional file 1: (S1, M5) [34, 39]. The metabolites by matching the exact m/z of the samples were annotated using the KEGG databases and human metabolome. The metabolites with *p-*value < 0.05 and VIP > 1 were considered significantly different.

### Microbial community analysis

To evaluate the differences in community structure, the gut microbial community of the CB-, CS-, and PS-fed larvae were analyzed. At the end of 30-day of feeding, fifteen larvae were randomly selected from the PS-, CS-, CB-fed groups and sterilized for 1 min with 75% ethanol, followed by washing twice with 0.9% (w/v) sterile saline [7]. Next, the guts were taken out and placed in a 2 mL centrifuge tube. Total DNA was extracted using the SPINeasy DNA Kit for Feces (MP Biomedicals, LLC, Singapore) based on the protocols. The PCR amplicon sequencing was performed as previously [10, 40]. Briefly, the hypervariable regions V3/V4 of the 16S rRNA gene were chosen for the PCR amplicon sequencing with the primers 338F/806R (F: ACTCCTACGGGAGGCAGCAG, R: GGACTACHVGGGTWTCTAAT) by using PCR amplifier (ABI GeneAmp® 9700). The unqualified sequences and adapters were removed from the raw sequencing data, and high-quality data existed in pair-end reads using the Illumina Hiseq 6000 platform. Microbial community structure, alpha diversity, hierarchical clustering, and the relative abundance of differential species were performed on Majorbio Cloud Platform (https://cloud.majorbio.com/). All groups (CB, PS, CS) were analyzed in four replicates.

### Metatranscriptome analysis

RNA extraction, Illumina sequencing and assembly, and functional genes annotation were conducted accordingly [41], details were presented in the Additional file 1: (S1, M6 &M7). The DEGs of comparisons of PS vs. CK (CB), and CS vs. CK groups were analyzed using the R package edgeR [42]. The Benjamini–Hochberg method was applied to adjust *P*-value of DEGs analysis with the false discovery rate (FDR) for multiple comparisons [43]. The selection criteria for DEGs were as follows: |log_2_^FC^|≥ 1, FDR ≤ 0.05, and transcripts per million (TPM) ≥ 50 in at least one sample. The assembled sequences of DEGs were compared to the NCBI Non-redundant Protein (Nr) database using the BLASTP algorithm based on a threshold of E-value < 10^−5^ for assigning predicted gene descriptions [44].

### RT-PCR validation

The seventeen DEGs from the PS and CS groups metatranscriptomic sequencing were confirmed via RT-PCR. cDNA was synthesized from total RNA using a reverse-transcription kit (RR047A, Takara) based on the manufacturer’s instructions. The primers for RT-PCR were designed by Primer3Plus (https://www.primer3plus.com), as shown in Additional file 1: (S1, Table S2). RT-PCR was performed using TB Green Premix Ex Taq™ II (RR820A, Takara) following the manufacturer’s protocol. The ribosomal protein L27a of *T. molitor* (TmL27a) was used as an endogenous gene [45]. RT-PCR included the following cycles: denaturation at 95 °C for 5 min, followed by 40 cycles of 95 °C for 15 s, 60 °C for 15 s, and 72 °C for 10 s. RT-PCR assays of each gene were performed in triplicate.

### Expression, purification, and functional assay of potential PS-/CS-degrading enzyme

To find potential enzymes that can degrade both PS and CS, according to the metatranscriptomic and RT-PCR results, we selected lac640 (LMCOs, k97_48640_gene_4_1) that was highly upregulated in both PS- and CS-fed groups. Previous research also reported laccase can degrade lignin and up-regulated in plastic degradation [32, 46]. Therefore, the enzyme was heterogeneously expressed in *E. coli* Rosetta (DE3) and purified as previously [46], details were shown in the Additional file 1: (S1, M8). The laccase activity of the protein Lac640 was assayed by detecting the oxidation of 2,2´-azino-bis (3-ethylbenzothiazoline-6-sulfonic acid) (ABTS) at 420 nm. The reaction volume of 3.2 mL included 3 mL of sodium acetate buffer (0.1 M) containing 10 mM CuSO_4_, 160 µL of ABTS (20 mM), and 40 µL of Lac640 solution. The optimal enzyme activities were detected with different Cu^2+^ concentrations (1–1000 mM), pH values (3 − 7), and temperatures (30 − 80 °C). The kinetics of Lac640 was measured at different initial ABTS concentrations. The effects of Lac640 in degrading PS/CS were detected in a solution including 0.1 M sodium acetate buffer (10 mM CuSO_4_), enzyme solution (0.1 mg/mL), and PS/CS powder or film at 40 °C for 72 h. FTIR and SEM analyses were performed to observe the changes in surface morphology and functional groups between Lac640-treated and untreated PS/CS samples [47]. After completion of the reaction, all samples were successively washed with 1% SDS, distilled water, and ethanol, then completely dried at 60 °C for FTIR analysis, or freeze-dried for SEM.

### Statistical analysis

The mean ± SD was used for all experimentally obtained data. Student’s t-test was used for two-group comparisons; for more than two groups, the significance of differences was analyzed using one-way ANOVA with the post hoc Tukey test. Significance was set at *P < 0.05, **P < 0.01, ***P < 0.001, and ****P < 0.0001. The significance of differences (*P* < 0.05) of each set of gut microbiota among the larval groups were analyzed using ANOVA with Benjamini–Hochberg FDR multiple tests [43].

## Results and discussion

### Survival of *T. molitor* larvae and consumption of PS and CS

**Fig. 1:**
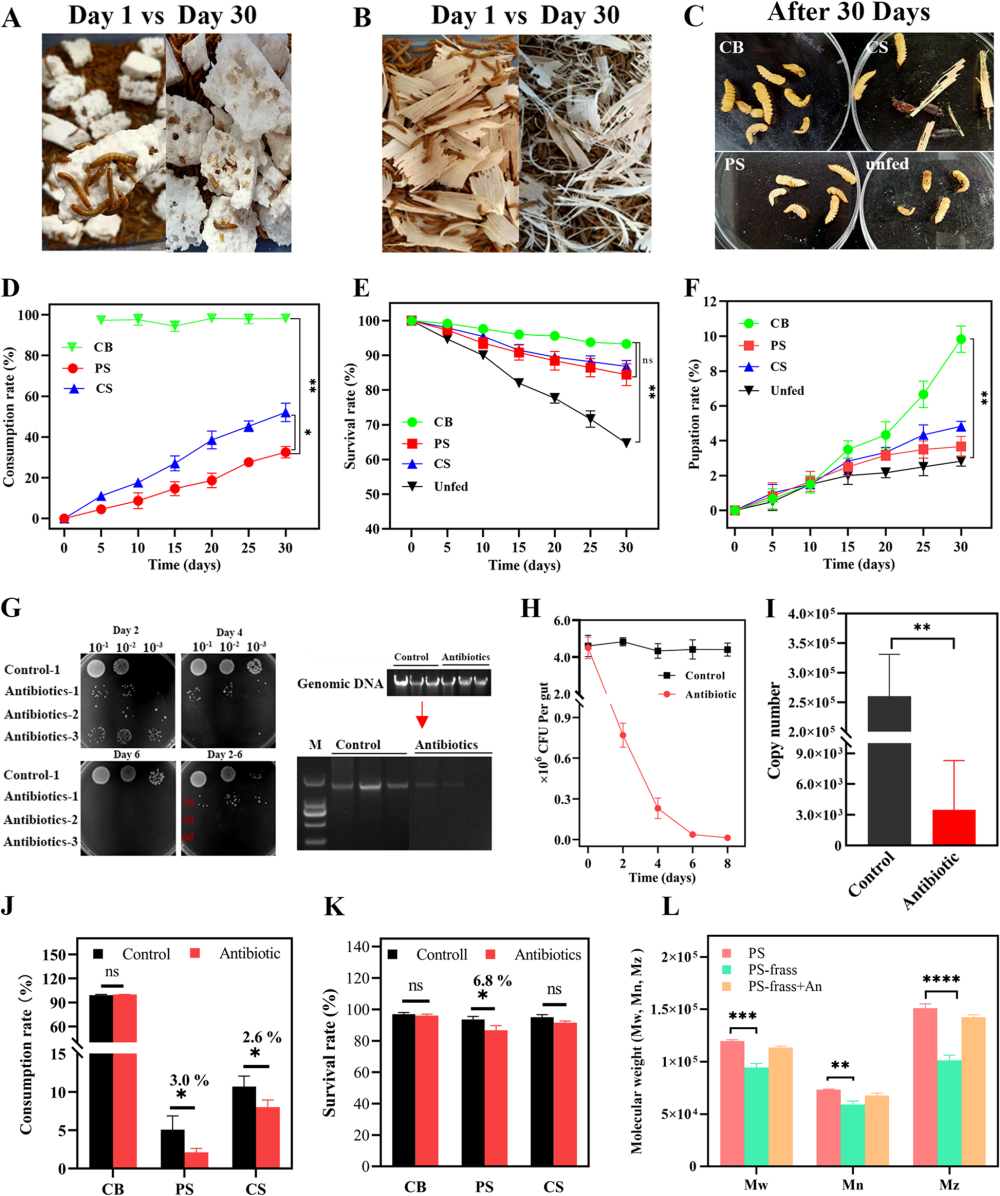
**A**-**C** Images of exposure of the yellow worms to PS and CS. **D**-**F** PS/CS consumption rates (**D**), survival rates (**E**), and pupation rates (**F**) of larvae fed with PS, CS, CB and unfed. **G** The gut bacteria of larvae after antibiotic treatment on TSA medium and electrophoretogram of conventional 16S rRNA PCR products. **H** Total visible colony counts of larvae gut flora within 8-days of antibiotic treatment and (**I**) the absolute quantification of 16S rRNA copies in guts of the control and antibiotic-treated groups. **J**-**K** Consumption and survival rates of antibiotics-treated and untreated larvae (control) fed with different feedstocks for 5 days. **L** The molecular changes (Mw, Mn and Mz) of PS residues extracted from frass of antibiotics-treated and untreated larvae fed PS vs. PS feedstock. Styrofoam (PS), Corn straw (CS), Cabbage (CB)

The mealworms were fed with PS and CS feedstocks (Fig. 1A, B), and the larvae were developed into pupae during the 30 days of the experiment (Fig. 1C). For mealworms initially fed 2 g of PS and CS, the total mass loss of PS was 0.67 ± 0.02 g and the CRs was 32.5 ± 1.5%, whereas the total mass loss of the CS was 1.05 ± 0.04 g and CRs was 52.0 ± 1.8% (Fig. 1D). The SRs of larvae fed with CB, PS, CS and unfed were 93.3%, 84.4%, 86.8% and 64.6%, respectively (Fig. 1E); their corresponding PRs were 9.8%, 3.7%, 4.8%, and 2.8% (Fig. 1F). The mass balance of PS and CS were estimated according to the CRs of feedstock, generated frass mass and frass extraction, and lignocellulose components in frass. The larvae were digested 63.5% (PS removal rate) of consumed-PS feedstocks, while digested 44.4%, 26.6%, and 33.3% of cellulose, hemicellulose, and lignin component of consumed-CS feedstocks, respectively (Additional file 1: S1, Table S3). The average weight of larvae was increased 23.3% in the CB-fed group, while PS-, CS-fed and unfed larvae were decreased 18.6%, 9.6%, and 26.8% of body weight, respectively (Additional file 1: S1, Fig. S2). GPC analysis showed that the Mw, Mn, and Mz of PS residues extracted from the PS-fed larvae frass were significantly reduced at a rate of 21.0%, 19.2% and 32.9% than the PS feedstocks (Fig. 1L). Specifically, Mw decreased from 120,000 to 94,466 Da, Mn decreased from 73,178 to 59,151 Da, and Mz decreased from 150,774 to 101,198 Da (Additional file 1: S1, Table S4).

The antibiotics test showed that gentamicin, vancomycin, and ampicillin had strong inhibition ability on the growth of larvae gut flora by making prominent halos whether used alone or in combination (Additional file 1: S1, Fig. S3). After 6-day of antibiotic treatment, there was no visible gut microbial colonies on TSA medium (Fig. 1G), the number of CFUs of the larval gut active flora was (0.1 × 10^6^) significantly decreased than control (4 × 10^6^) (Fig. 1H). The RT-qPCR results also showed that the copy number of gut microbes from the antibiotic-treated larvae (4 × 10^3^) was significantly decreased compared with the control (2.5 × 10^5^) (Fig. 1I). The PS/CS consumption rates of antibiotic-treated larvae were decreased 3.0% and 2.6% compared with the control, respectively (Fig. 1J). Similarly, the SRs of antibiotic-treated larvae fed with PS and CS were slightly decreased 6.8% and 3.0% than that of the control after 5 days (Fig. 1K). Antibiotic treatment had no significant impact on the SRs and CRs of CB-fed larvae. The GPC results showed that Mw, Mn and Mz values of PS residues extracted from frass of antibiotic-treated larvae fed with PS were also slightly decreased (Mw: 6065 Da, Mn: 5392 Da, Mz: 8339 Da) than raw PS feedstocks (Fig. 1L) (Additional file 1: S1, Table S4).

The PS consumption rate (32.5%) of this study was consistent with the range of rates (31%–41.5%) reported previously by other researchers [7, 9]. The CS consumption of larvae (52.0%) was lower than that in a previous study which was 90% [48], this disparity can be attributed to different lignocellulose contents or the hardness of CS materials. The SRs of PS- and CS-fed larvae were ~ 10% higher than that of the unfed group (*P* < 0.01), but there was no significant difference between CB-, CS- and PS-fed groups (*P* > 0.05) (Fig. 1E), which was also supported by previous studies [9, 48]. The number of pupae is widely used to evaluate the healthy development of larvae [36], the PRs of larvae fed PS and CS were higher than the unfed group, but lower than the CB-fed group, during the 30 days. The mass balance results revealed that the larvae cannot remove or digest all consumed-PS/-CS feedstocks, and partially consumed-feedstocks excrete with feces, which was consistent with previous reports [34, 37, 48]. The CB feedstocks conversion rate by larvae was higher than PS and CS feedstocks, this resulting in an increased weight of CB-fed larvae than other diets groups. Except for CB-fed larvae, the PS-, CS-fed and unfed larvae lost their weights, the unfed group was the one with the higher weight loss (Additional file 1: S1, Fig. S2). In a previous study, the average weight of mealworms fed with only PS or PVC foam was also decreased after 31-day tests [12, 49], which was consistent with the present study. Besides, the mealworms fed with CS diet increased their body weights by 2.6% [48], but our studies observed that the CS-fed larvae lost weight, this may be attributed to the components and structure of CS materials. These findings imply that larvae can obtain some energy from PS and CS for survival, but lack sufficient nutrients for healthy growth when unfed or only fed PS and CS. Several studies have reported that the Mn and Mw of PS residues extracted from only PS-fed mealworm feces were lower than that of the raw PS feedstocks, and the reduced range of Mw and Mn were 7.5 ~ 38.9% and 8.75% ~ 34.6%, respectively [7, 9, 34, 49, 50]. However, the variable reduction rate depends on the differential molecular weight of the raw PS materials. In our study, the Mw and Mn of PS feedstocks were 120.0 kDa and 73.2 kDa, and the reduced rate were 21% (25.1 kDa) and 19.2% (14.0 kDa), respectively. For the Mw and Mn of PS materials similar with our study, their reduction rates of Mn and Mw in the PS-fed larvae feces were 20.8% (124.2 kDa ~ 98.3 kDa) and 20.2% (40. 4 kDa ~ 32.3 kDa) [7]. Nonetheless, the depolymerization observed in PS-fed larvae was within the range of previous observations.

The antibiotic treatment impaired the ability to digest PS due to suppression of the larval gut microbiota, thereby decreasing the SRs of mealworms compared with that of the control group. Previous studies reported that the larval gut microbes depressed by antibiotic resulted inhibited PS degradation, but not LDPE plastic [7, 36]. Our findings observed the Mw, Mn and Mz of PS residues from antibiotic-treated larvae frass were slightly decreased compared with the raw PS foam, indicating that host enzymes also contributed in the PS depolymerization. This synergetic role of the mealworms host and its gut microbiome for plastic biodegradation has been proven [51]. However, there has no study on the role of larval gut microbes in the CS degradation. Our antibiotics test showed that the degradation of CS was not significantly dependent on gut microbiota as PS foam, but antibiotic treatment had slight effect on CS consumption rates of larvae compared with the antibiotic-untreated larvae.

### Depolymerization and comparative metabolomic analysis of PS and CS

**Fig. 2:**
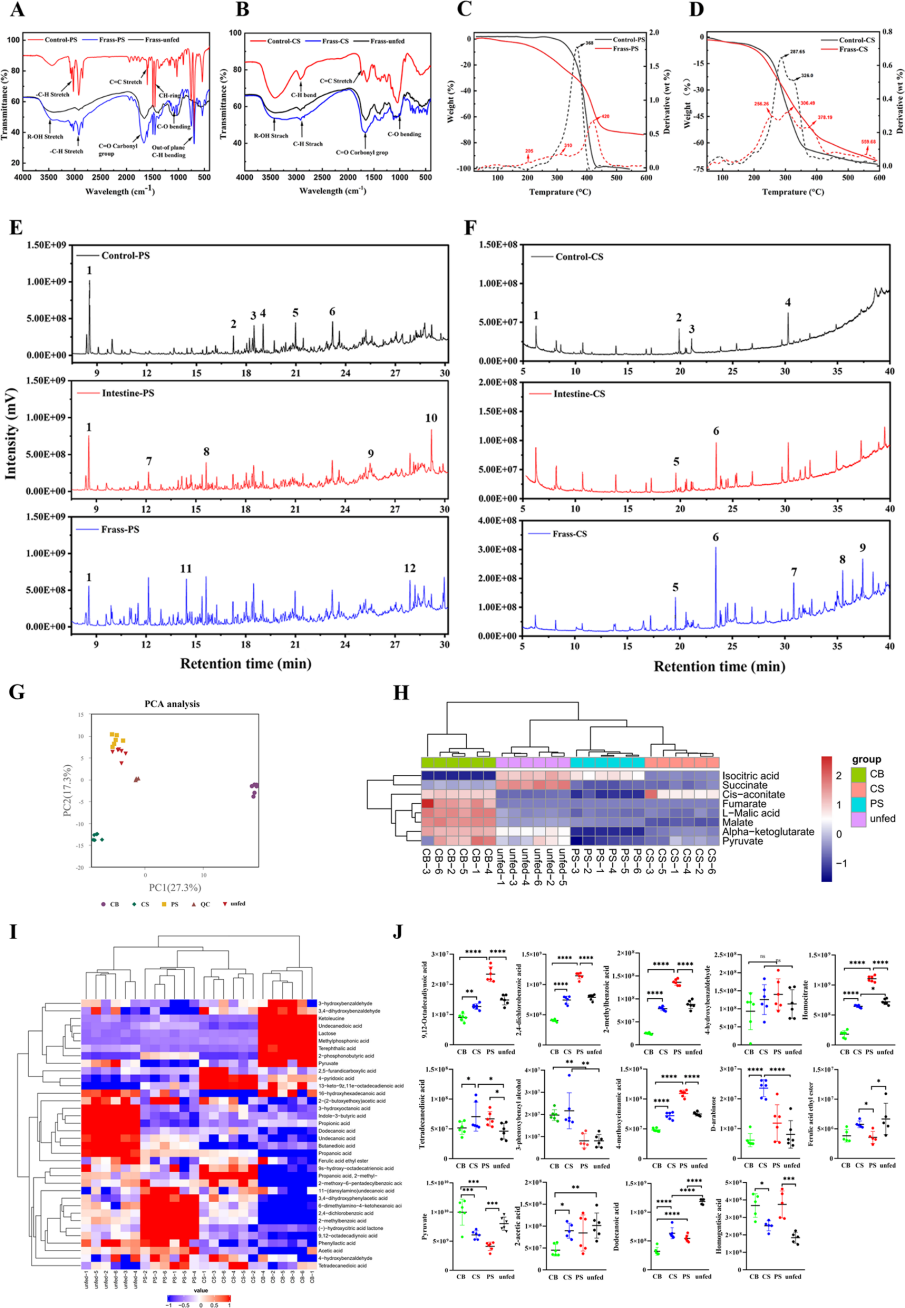
**A**, **B** FTIR spectral analysis of raw PS/CS (control) and frass samples from PS-/CS-fed larvae. **C**, **D** TGA analysis of frass from PS-/CS-fed larvae [solid lines represent weight curve (left axis), whereas dashed lines represent derivative curve (right axis)]. **E**, **F** GC–MS analysis of the frass and intestines of PS-/CS-fed and control PS/CS samples. Compounds in figure (**E**): 1. benzene, 1,3-dimethyl-(C_8_H_10_); 2. tetradecane (C_14_H_30_); 3. cyclohexene, 1-methyl-4-(C_10_H_16_); 4. 2,4-di-tert-butylphenol (C_14_H_22_O); 5. octadecane, 3-ethyl-5-(2-ethylbutyl) (C_26_H_54_); 6. phthalic acid, bis(7-methyloctyl) ester (C_26_H_42_O_4_); 7. undecanoic acid (C_11_H_22_O_2_); 8. pentadecanoic acid (C_15_H_30_O_2_); 9. oleic acid, 3-(octadecyloxy)propyl ester (C_39_H_76_O_3_); 10. hexadecanoic acid, ethyl ester (C_18_H_36_O_2_); 11. 3,6-octadecadiynoic acid, (C_19_H_30_O_2_); 12. 11,14-eicosadienoic acid, methyl ester (C_21_H_38_O_2_); compounds in figure (**F**): 1. tetratetracontane (C_44_H_90_); 2. cyclohexane, 1,3,5-trimethyl-2-octadecyl-(C_27_H_54_); 3. eicosane, 2-methyl-(C_21_H_44_); 4. phenol, 2,2-methylenebis[6-(1,1-dimethylethyl)-4-methyl-(C_23_H_32_O_2_); 5. hexadecanoic acid, methyl ester (C_17_H_34_O_2_); 6. 9-octadecenoic acid, methyl ester (C_19_H_36_O_2_); 7. tetradecanoic acid, methyl tetradecanoate (C_15_H_30_O_2_); 8. decanoic acid (C_40_H_64_O_8_); 9. hexadecanoic acid, octadecyl ester (C_34_H_68_O_2_). **G** PCA analysis for metabolome of the gut of mealworms under CB-, PS-, CS-fed and unfed conditions (*n* = 6); Note: QC samples are made by mixing the samples to be tested in equal quantities, used to monitor, and evaluate the stability of the system and the reliability of the experimental data. **H** Cluster heat maps of differential metabolites in the KEGG pathway. **I** Hierarchical cluster heat maps of significantly different metabolites. **J** Relative abundance of key metabolites of PS and CS degradation

FTIR spectra of the frass from PS-fed larvae showed new functional groups of C = O (1700 cm^−1^) and C-O stretches (1050 − 1150 cm^−1^) (Fig. 2A). The peak of R-OH (2500–3500 cm^−1^) stretching of the hydrophobic group was broader than that in the control raw PS. Similar FTIR spectra appeared in the CS-fed larvae frass, including oxygen- and hydrophobic group associated peaks C-O (800–1250 cm^−1^), C = O stretches (1500–1800 cm^−1^), and alcohol groups (R-OH stretching, 3200–3700 cm^−1^) (Fig. 2B). TGA analysis indicated that the weight loss of control PS was 98.1% at 280 °C–450 °C, with the maximum decomposition rate at 368 °C (Fig. 2C); meanwhile, the frass from PS-fed larvae contained three decomposition stages, with a total of 70% weight loss occurring at 100 °C–500 °C. The control CS sample had two decomposition stages: the weight loss in the first stage was 46.2% at 200 °C–310 °C, and 32.5% weight loss occurred at 310 °C–410 °C in the second stage (Fig. 2D). In contrast, the frass from CS-fed larvae was decomposed in four stages, with corresponding weight loss of 19.5%, 21.4%, 11.8%, and 4.7% at 256 °C, 306 °C, 378 °C, and 559 °C, respectively. The SEM images revealed that the surface of CS/PS feedstocks and excreted feces by larvae fed with PS and CS were different, suggesting that the PS and CS were digested and converted into another monomers by larvae **(**Additional file 1: S1, Fig. S4). The EDS mapping showed the frass of PS- and CS-fed larvae containing C, O and N, indicating the consumed PS and CS by larvae were converted to carbon–oxygen substance (Additional file 1: S1, Fig. S4).

To determine the degradation intermediates of PS and CS, the gut and frass samples from the PS-, CS-, CB-fed and unfed larvae were profiled by GC–MS (Additional file 1: S1, Table S5). Long-chain fatty acids, such as undecanoic acid (C_11_H_22_O_2_), oleic acid, 3-(octadecyloxy) propyl ester (C_39_H_76_O_3_), 3,6-octadecadiynoic acid (C_19_H_30_O_2_), and hexadecanoic acid (C_18_H_36_O_2_), were found in the frass or intestinal samples of PS-fed larvae (Fig. 2E), whereas the relative abundances of benzene, 1,3-dimethyl-(C_8_H_10_), cyclohexene (C_26_H_52_), and phthalic acid (C_26_H_42_O_4_) were decreased. Similarly, in the frass or intestinal samples of CS-fed larvae, hexadecanoic acid (C_18_H_36_O_2_), 9-octadecenoic acid (C_19_H_36_O_2_), and tetradecanoic acid (C_15_H_30_O_2_) were also found (Fig. 2F), along with decreases of complex long-chain hydrocarbons or ring structures such as eicosane, 2-methyl-(C_21_H_44_), cyclohexane (C_26_H_52_), and phenol,2,2-methylenebis (C_23_H_32_O_2_). In addition, the gut samples of CB-fed and unfed larvae presented some similar peaks, as well as the frass samples of CB-fed and unfed larvae; this may be due to gut tissue or frass characterization. Despite the similarities in the gut or frass samples of CB- and unfed, the results of the GC–MS analysis were distinct from those obtained from the PS- and CS-fed larvae samples (Additional file 1: S1, Fig. S5).

Comparative metabolomic analysis was performed to characterize differential metabolites of CB-, PS-, CS-fed and unfed larvae by using LC–MS/MS. In total, 2489 metabolites were identified in the gut of larvae fed with CB-, PS-, CS-fed and unfed. Most of the metabolites located in organic acids and derivatives (25.39%), lipids and lipid-like molecules (21.54%), organoheterocyclic compounds (13.218%) and benzenoids (11.089%) (Additional file 1: S1, Fig. S6A). PCA (Principal Component Analysis) analysis of gut samples metabolites of larvae fed with CB, PS, CS feedstocks and unfed were significantly distinct from each-other (Fig. 2G). The hierarchical clustered heat maps of significantly differential metabolites showed that the PS and CS clustered one small branch, indicating PS and CS metabolites expression patterns were similar (Fig. 2H-I). The volcano map analysis showed the metabolites of PS- and CS-fed larvae were differentially expressed compared with the control group CB-fed and unfed group (Additional file 1: S1, Fig. S6B). A total of 706, 655, 561 and 581 differential metabolites were detected in the comparisons between PS vs. CB, CS vs. CB, PS vs. unfed, CS vs. unfed, respectively (Additional file 1: S2 & S3). Among them, 321 and 296 metabolites were upregulated in PS- and CS-fed groups compared with the CB-fed group, while 154 and 182 metabolites were upregulated in PS- and CS-fed groups compared with the unfed group. The relative abundance of differentially expressed metabolites, such as 9,12-octadecadiynoic acid, 2-methylbenzoic acid, 2,4-dichlorobenzoic acid, 4-hydroxybenzaldehyde and homocitrate were higher in PS-fed larvae than another groups (Fig. 2J). The tetradecanedioic acid, 3-phenoxybenzyl alcohol and D-arabinose were enriched in the CS-fed group, whereas the high relative abundance of pyruvate and 2-acetic acid in the CB or unfed group.

In previous studies, peaks of C = O (1700 cm^–1^) and C-O (1050–1200 cm^–1^) stretching appeared and R-OH (2600–3600 cm^−1^) stretching broadened in the FTIR spectra of the frass from PS-/CS-fed larvae [9, 23]. In this study, we also observed similar changes in the frass of PS-/CS-fed larvae, this illustrates of the PS and CS were oxidized by larvae, resulting in increased hydrophilicity compared to the control PS/CS. The TGA of control PS/CS and frass of PS-/CS-fed larvae showed obvious changes in chemical composition. The PS foam lost 98.1% of its weight at the one stage, whereas the frass of PS-fed larvae only lost 70% of its weight with three additional stages under the same heating condition, which was consistent with previous results [7]. The frass of CS-fed larvae had more decomposition stages than that of the control CS; the total weight loss of the control CS was 74.5% at 200 °C–450 °C, whereas the frass from CS-fed larvae was 52.8% of the weight. This suggests that the detritus components of PS- and CS-fed larvae were converted to degradation products after passing through the gut. Previous GC–MS analysis reported that oleic acid and octadecanoic acid as the intermediates of PS metabolism in *Galleria mellonella* larvae [10], whereas hexadecanoic acid, 9-octadecenoic acid, and benzoic acid (C_6_H_5_COOH) were detected as CS intermediates [38]. Exceptionally, D-( +)-galactose (C_16_H_21_NO_10_) was detected in the frass of CS-fed larvae (Additional file 1: S1, Table S5), indicating that lignocellulose was degraded into monosaccharides [52]. In our comparative metabolomic analysis, we observed PS-related metabolites, such as 9,12-octadecadiynoic acid, 2,4-dichlorobenzoic acid, 4-hydroxybenzaldehyde, 2-methylbenzoic acid and homocitrate etc., which has been discovered in the PS degradation metabolites in *C. mellonella* larvae [39]. The ferulic acid ethyl ester and 4-methoxycinnamic acid are known as lignin degradation intermediates [53, 54], whereas D-arabinose and lactose are the degradation byproducts of hemicellulose and cellulose [55]. These intermediates were also detected in the present metabolomic analysis with high relative abundances in CS- or CB-fed larvae. Some fatty acid or amino acid metabolites exhibited higher abundances in the unfed group, which might be produced by stored energy in the body or by eating dead larvae or molt. Our GC–MS analysis also showed similar PS and CS degradation metabolites (e.g., 9,12-octadecadiynoic acid and tetradecanedioic acid) with that of the metabolomic analysis. This evidence revealed the potential degradation metabolism of PS and CS polymers.

### Responses of gut microbiota to the different diets

**Fig. 3:**
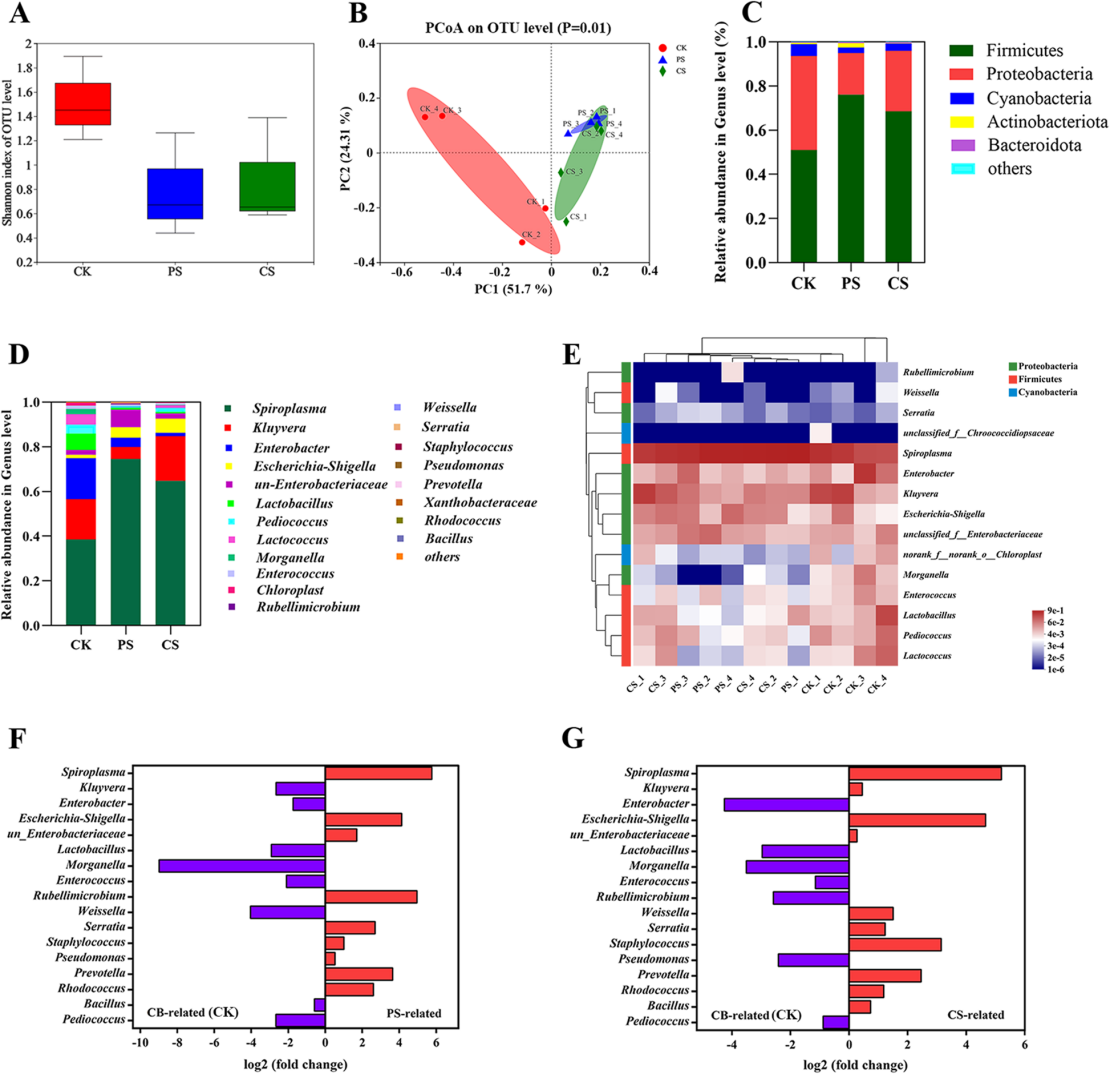
Gut microbial community analysis of larvae fed with different diets. **A** Shannon index and **B** PCoA analysis of gut microbial community (OTU level) from larvae fed PS, CS, and CB (CK). **C**-**D** Relative abundances of larval gut microbes with different diets at the (**C**) phylum and (**D**) genus level. **E** Hierarchical clustered heatmap analysis of larval gut microbes at the genus level. **F**, **G** Differential abundance analyses at the OTU level [PS-fed vs. CK-fed (**F**); CS-fed vs. CK-fed (**G**)]. The direction of log2 (fold change) implies that the OTU is highly related to each diet

The microbial diversity of *T. molitor* larvae fed with PS, CS, and CB diets were analyzed to determine the predominant gut microbes (Fig. 3). A total of 669,074 sequences were achieved from the three groups, with good coverage of 99% (Additional file 1: S1, Table S6). The Shannon index showed lower species richness of the gut microbiome of PS- and CS-fed larvae than in the CK (CB-fed larvae) group (Fig. 3A). A principal coordinate analysis (PCoA) based on the OTU revealed that the microbial composition of PS and CS group was distinct from that of CK group, but there was no clear distinction between PS and CS group (Fig. 3B). Furthermore, hierarchically clustered heatmap analyses of CB-, PS-, and CS-fed larvae at the genus level were cluster I comprised CK-4 and CK-3, whereas cluster II had two branches, one including control samples CK-2 and CK-1, another included CS and PS group (Fig. 3E). The gut microbial community of PS and CS group was significantly separated from that of the CB-fed group, but the PS and CS group was clustered into one branch. At the phylum level, the relative abundances of Firmicutes in PS (81.2%) and CS (73.5%) groups were greater than in the CK (56.1%), whereas the relative abundances of Proteobacteria of PS (18.7%) and CS (26.4%) groups were lower compared to the CK group (42.5%) (Fig. 3C). At the genus level, *Spiroplasma*, *Kluyvera*, and *Enterobacter* were the predominant genera among the larval gut microbiota in all diet groups (Fig. 3D). Unclassified *Enterobacteriaceae*, *Serratia*, and *Rubellimicrobium* were dominant in the PS-fed group, whereas *Staphylococcus* and *Weissella* were enriched in the CS-fed group. The relative abundances of *Spiroplasma* (PS: 74.5%, CS: 64.8%) and *Escherichia–Shigella* (PS: 4.8%, CS: 6.3%) increased in both PS- and CS-fed groups compared with those in the CK group (38.5%, 1.4%). To further evaluate the particular OTUs related to the PS and CS diets, differential abundance analyses of the PS vs. CB group and CS vs. CB groups were conducted (Fig. 3F, G). *Rubellimicrobium* sp. and *Pseudomonas* sp. exhibited significant differences in the gut of PS-fed larvae, whereas *Kluyvera* sp., *Weissella* sp., and *Bacilius* sp. maintained high abundances in the gut of CS-fed larvae. Furthermore, *Spiroplasma* sp., *Escherichia–Shigella* sp., unclassified *Enterobacteriaceae* sp., *Serratia* sp., *Staphylococcus* sp., *Prevotella* sp., and *Rhodococcus* sp. exhibited higher abundances in both PS- and CS-fed groups than in CB-fed larvae.

The alpha diversity results indicated that the richness of gut flora was dropped after PS and CS feeding compared with the CB diet, but species diversity was not significantly affected. The PCoA and hierarchical clustered heatmap analysis suggested that most members of the gut microbial community of larvae did not differ dramatically between the PS and CS diet groups, indicating that the gut microbial community structures of PS- and CS-fed larvae were similar. Previous studies reported that the phylum Firmicutes and the two genera *Spiroplasma* and *Escherichia–Shigella* presented at high abundance in the gut microbiota of PS-fed larvae, and *Spiroplasma* helped to protect against entomopathogens [40]. Most microorganisms belonging to the *Enterobacteriaceae* family were strongly associated with plastic biodegradation [9, 12, 49, 56]. The *Serratia* sp. wsw isolated from the gut of *Plesiophthalmus davidis* larvae can degrade PS plastics [57], and also reported it associated with CS and rice straw degradation in the mealworms gut [23]. The genus *Rubellimicrobium* was predominant in plastic co-amended soil [58]. *Pseudomonas* is a typical plastic-degrading microorganism, such as *P. aeruginosa*, *P. syringae*, and *P. putida*, which exhibited efficient plastic degradation capabilities [5, 59]. In addition, the *Pseudomonas* also related to PS degradation in the gut of mealworms and land snails *Achatina fulica* [13, 40]. These findings suggested that *Serratia* sp., *Rubellimicrobium* sp., and *Pseudomonas* sp. were associated with PS degradation in mealworms. The differential abundance analysis revealed that *Staphylococcus*, *Weissella*, *Kluyvera*, and *Bacillus* were present at significantly different abundances in the CS-fed group compared with the other groups (Fig. 3D). Among these, the genus *Staphylococcus* from the gut of *Macrotermes nigeriense* termites decomposed lignocellulose due to the secretion of lignin peroxidase [60], whereas *Kluyvera* sp. with xylanase and cellulase activity involved in lignocellulose degradation [61]. Additionally, the relative abundance of *Kluyvera* sp. was enriched in the gut of PP-fed mealworms [56]. However, there are no available report regarding *Weissella* related to plastic or lignocellulose degradation; this genus requires further exploration of its lignin or plastic degradation ability. The OTUs analysis revealed that unclassified *Enterobacteriaceae* sp., *Serratia* sp., *Staphylococcus* sp., *Prevotella* sp., and *Rhodococcus* sp. were associated with both PS and CS diets (Fig. 3F, G). These strains reportedly have the potential to degrade plastic and lignocellulose [6, 57, 60, 62], contributing to improve the degradation efficiency of plastic and lignocellulose in the larval gut. These findings suggest that the gut microbes of larvae were restructured by the different diets, resulting in a similar community structures upon feeding on PS and CS.

### Metatranscriptomic analysis of mealworms fed PS and CS

Metatranscriptomic sequencing was applied to determine the metabolically active bacteria and the mechanism of plastic and lignocellulose degradation. A total of 87,468,710 clean reads with 11.3G clean bases, 89,290,414 clean reads with 11.5G clean bases, and 89,150,052 clean reads with 11.4G clean bases were obtained from the CK (CB), PS, and CS groups, respectively (Additional file 1: S1, Table S7). Overall, there were 28,413, 31,636, and 46,494 unigenes with N_50_ (N_90_) lengths of 1138 bp (399 bp), 1148 bp (392 bp), and 1421 bp (423 bp) in the CK, PS, and CS groups. There were most unigenes with lengths of 1–700 bp/701–1400 bp in all samples (Additional file 1: S1, Fig. S7). The relative abundance of larval gut microbiota was changed at the RNA level after feeding on the different diets. At the phylum level, PS and CS exposure altered the structure of larval gut microbiota (Additional file 1: S1, Fig. S8A). Specifically, the relative abundances of Tenericutes, Firmicutes, and Bacteroidetes were increased, whereas Proteobacteria and Cyanobacteria were decreased in the PS- and CS-fed groups, which was consistent with the previous study [40]. The genus level analysis showed that *Spiroplasma* was predominant in the gut of mealworms in all the diet groups (Additional file 1: S1, Fig. S8B). These results were almost consistent with the Illumina sequencing of 16S rRNA sequences (Fig. 3C, D). *Streptococcus*, *Halobacterium*, *Bacillus*, *Pediococcus*, *Desulfurobacterium*, and *Aspergillus* exhibited high abundance in the PS- and CS-fed larvae (Additional file 1: S1, Fig. S8C, D). These genera are known as plastic- or lignocellulose-degrading bacteria or fungi [23, 32, 49, 63], and these microbes might be related to PS and CS degradation in the larval gut.

**Fig. 4:**
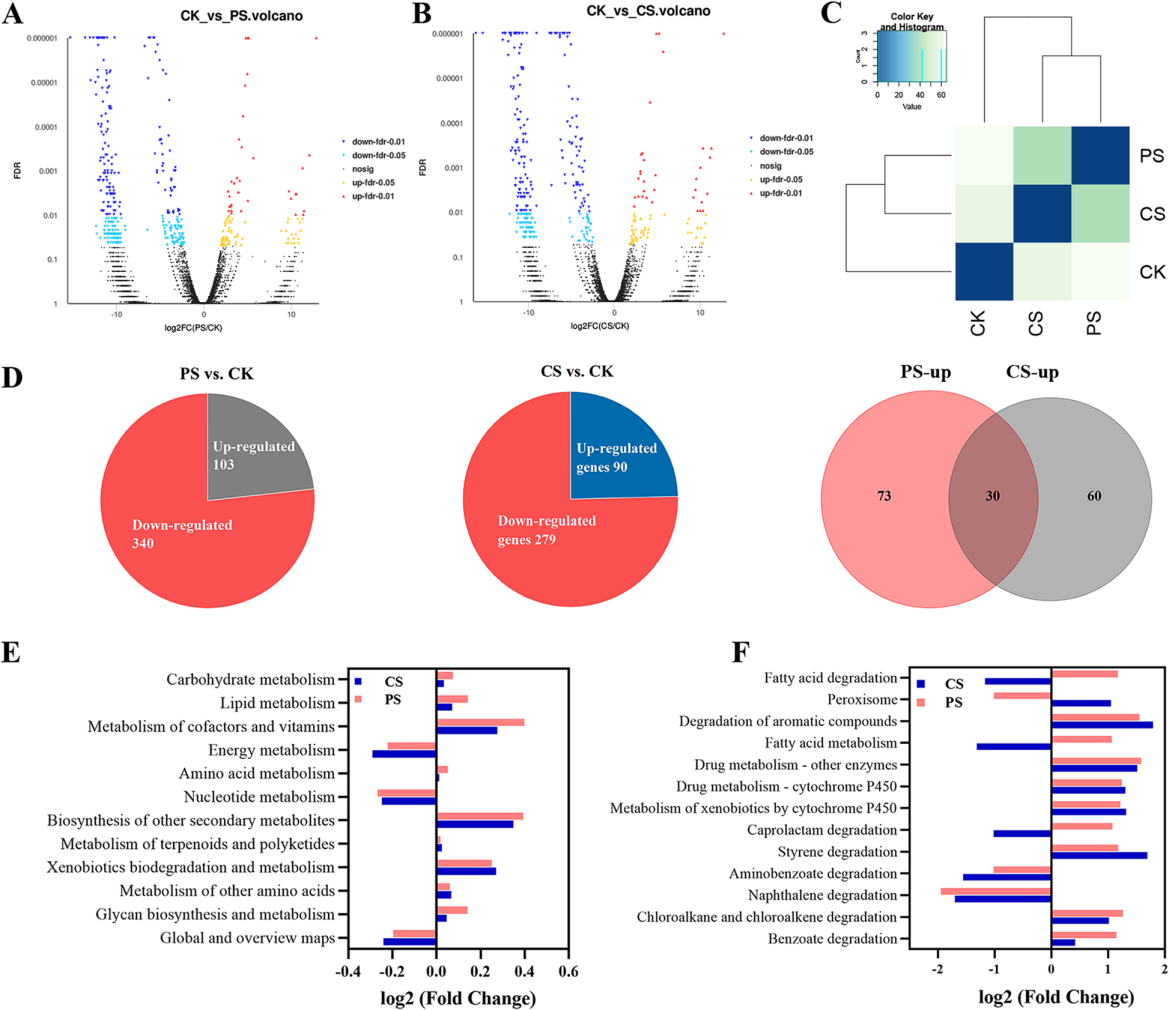
The DEGs analysis of larvae fed with different diets by metatranscriptomic sequencing. **A**, **B** Volcano map of the DEGs in response to PS and CS. **C** Heat map of DEGs in response to three different diets (PS, CS and CB). **D** Venn diagram of up- or down-regulated gene responses to PS or CS. (E, F) KEGG pathway enrichment analysis of DEGs in response to PS and CS at KEGG level 2 (**E**) and level 3 (**F**)

In the comparative PS vs. CK and CS vs. CK metatranscriptomic libraries, 8082 and 8186 of DEGs were obtained, respectively. A total of 443 and 370 genes [log_2_(FC) ≥ 1 and FDR ≤ 0.05, *P* ≤ 0.05] showed significant changes after feeding with PS and CS, respectively (Fig. 4A, B). Among them, 103 and 91 DEGs were upregulated, 340 and 279 genes were downregulated in the PS-/CS-fed groups, respectively, whereas 30 DEGs were upregulated in both PS- and CS-fed groups (Fig. 4D). A heatmap of the DEGs of the three different diet groups generated two clusters; PS and CS clustered together, whereas CK clustered alone, indicating that the gene responses of larvae were similar against the PS and CS diets (Fig. 4C). According to the KEGG gene annotation, the dominated relative abundances of DEGs in the PS- and CS-fed groups were mainly located in clusters of carbohydrates, lipids, cofactors and vitamins, biosynthesis of other secondary metabolites, and xenobiotic metabolism compared with the CK group in KEGG level 2 (Fig. 4E). Metabolism of regarding the annotated xenobiotic biodegradation: benzoate degradation, styrene degradation, chloroalkane and chloroalkene degradation, metabolism of xenobiotics by cytochrome P450, and drug metabolism other enzymes; and global and overview maps: degradation of aromatic compounds were enriched in both PS- and CS-fed groups (Fig. 4F). The alcohol dehydrogenase [ADH; EC:1.2.1.10], cytochrome P450 family 6 [CYP6; EC:1.14.-.-], alkylglycerol monooxygenase [AGMO; EC:1.14.16.5], aldehyde dehydrogenase family [ALDH; EC:1.2.1.3], peroxidase [PO; EC:1.11.1.7], superoxide dismutase (Fe–Mn) family [SOD; EC:1.15.1.1], and long-chain-fatty-acid–CoA ligase [ACSL; EC:6.2.1.3] were found in the DEGs of PS-and CS-fed groups (Additional file 1: S1, Table S8). As typical lignocellulose- and plastic-degrading enzymes, CAZymes, such as laccase-like multicopper oxidase [AA1; EC 1.10.3.-], carboxyl esterase [CE10; EC 3.1.1.3], lipase [CE1; EC 3.1.1.-], endo-beta-1,3(4)-glucanase [GH12; EC 3.2.1.6], acetyl xylan esterase [CE1; EC 3.1.1.72], and feruloyl esterase [CE1; EC 3.1.1.73] were found in the PS-/CS-fed larval groups. Collectively, the genes involved in lignin and plastic degradation were classified into the AA or CE family, whereas hemicellulose and cellulose were classified into the GH/CE families (Additional file 1: S1, Fig. S9).

The corresponding bacterial species and their relative abundances for those enzyme sources in the 16S rRNA and metatranscriptomic taxonomy were analyzed by Nr database (Additional file 1: S1, Table S9 & Fig. S10). Although most enzymes were derived from the host genes, eight enzymes were resulted from larval gut microbes, which belongs to Enterobacteriaceae family, such as *Kluyvera*, *Serratia*, and *Bacillus*. Spatially, the laccase-like multicopper oxidase CueO (k97_48640_gene_4-1, Lac640) belonged to the *Kluyvera* genus. The relative abundance of *Kluyvera* in 16S rRNA gut microbes was higher in the CK (0.17) and CS (0.18) groups compared with PS (0.043) group, whereas metatranscriptomic taxonomy analysis observed a significant enrichment of *Kluyvera* in the PS (0.009) and CS (0.015) groups compared with the CK group (0.005). This bacterium and the laccase-like multicopper oxidase CueO (Lac640) maybe highly related to PS and CS degradation.

Previous studies reported that acetyl-CoA acyltransferase, carboxylesterase, CYP6, and CPY9 enzymes were overexpressed in LDPE-/PS-fed mealworms [64]. The relative abundances of alcohol dehydrogenase and aldehyde dehydrogenase were increased in PE-fed group compared with that in the honeycomb-fed and starvation larvae *G. mellonella* [65]. Numerous studies have reported that laccase is an important enzyme for lignin degradation in termites [66, 67]. The above enzymes LMCOs, SOD, and AGMO were also detected in both PS- and CS-fed groups (Additional file 1: S1, Table S8). Several microbial enzymes including Lac-, Lip-, and Mn- peroxidases can degrade plastic and lignin [25, 68]. Additionally, CYP4/6, SOD, and peroxidase were reported to be related to lignin oxidation [69, 70], whereas acetyl xylan esterase (AXE1) and endo-beta-1,3(4)-glucanase (Eb-G) can hydrolyze wheat straw or celluloses [66, 71]. Our transcriptomic analysis also found a series of oxidase (Peroxidase, LMCOs, SOD, CYP4/6, AGMO) and hydrolase (Carboxylesterase, Lipases and AXE1) related to the degradation pathways of lignocellulose and PS.

Previous studies confirmed fatty acid-, drug-, and xenobiotic-degradation metabolism enriched in PS- and CS-fed mealworms [64], whereas drug metabolism-other enzymes, fatty acid biosynthesis and xenobiotic biodegradation pathways enriched in lignin degradation strain *Aspergillus sydowii* MS-19 [72]. Our findings showed that benzoate degradation, styrene degradation, chloroalkane and chloroalkene degradation, metabolism of xenobiotics by cytochrome P450, drug metabolism, and degradation of aromatic compounds were enriched in the DEGs of PS- and CS-fed mealworms. (Fig. 4F), which was in accordance with previous studies [64, 65, 72]. These results suggest that the metabolism of long-chain fatty acids, xenobiotics, styrene, and aromatic compounds degradation were related to the plastic and lignin degradation metabolism, and the above-mentioned enzymes may participate in these pathways.

### Analysis of the potential metabolic pathways of PS/CS in *T. molitor* larvae

**Fig. 5:**
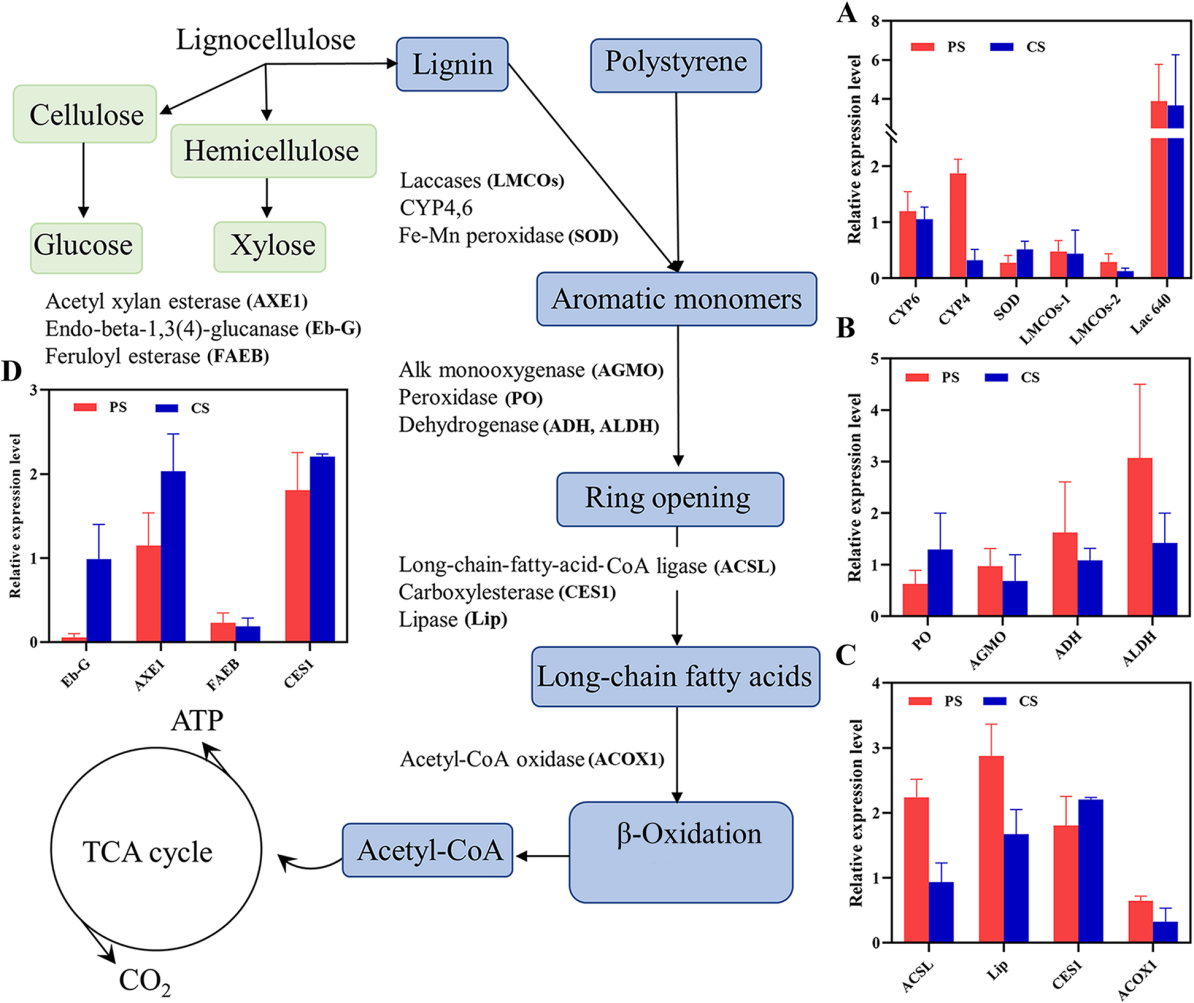
A proposed metabolic pathways of lignocellulose (CS) and PS plastic degradation in mealworms, and validation of the expression levels of related PS- and CS-degrading enzymes by RT-PCR (**A**-**D**). The 60S ribosomal protein 27a (Tml27a) of *T. molitor* was used as a reference gene; expression levels were calculated by the 2^−ΔΔCt^

The PS and CS are metabolized by the larvae, and produce energy for growth and development, but how the larvae metabolize these refractory synthetic plastic and natural polymers? this needs to further investigation. The metatranscriptomic analysis revealed that the DEGs responses, some metabolic pathways in KEGG, and enriched enzymes were similar in both PS- and CS-fed groups. Moreover, the GC–MS and metabolomic analysis revealed some similar intermediates in the gut or frass samples of PS- and CS-fed larvae. Based on present and previous results, we assumed that the degradation of these two polymers in the larvae have overlapped metabolic pathways, and therefore, we proposed the potential PS and CS metabolic pathways in the larvae (Fig. 5). The plastic- and lignocellulose-degrading enzymes from the DEGs were validated by RT-PCR, their expression levels in the PS-/CS-fed groups were calculated by the 2^−ΔΔCt^ (Fig. 5A-D). The expression levels of CYP6/4, Lac640, and SOD were upregulated in the PS- and CS-fed groups, especially, the gene Lac640 were two times higher rather than other enzymes (Fig. 5A). These enzymes can oxidize the side chain or break C–C bonds of PS and lignin, and convert them into aromatic residues or monomers. The monomer of PS is styrene, and the styrene degradation pathway was found in the KEGG analysis of the PS-fed group (Additional file 1: S1, Fig. S11). After the oxidation of PS and lignin, the aromatic benzene ring was cleaved driven by alkylglycerol monooxygenase, aldehyde dehydrogenase, alcohol dehydrogenase, and peroxidase (Fig. 5B). Then, the products were further hydrolyzed to generate long-chain fatty acids via carboxylesterase (CES1), long-chin fatty acid-CoA ligase (ACSL) and lipase, and these enzymes were upregulated in both PS- and CS-fed groups (Fig. 5C). These fatty acids are subsequently stored in the host or undergo β-oxidation for the TCA cycle and produce metabolic energy. The metabolism of lignocellulose involves the lignin, hemicellulose, and cellulose degradation pathways. After lignin modification, expos of cellulose and hemicellulose fibers were converted into glucose or xylose by AXE1, FAEB, CES1, and Eb-G, respectively. The enzymes AXEI and Eb-G exhibited higher expression levels in the CS-fed group than PS group (Fig. 5D), indicating that they are involved in cellulose and hemicellulose degradation.

The PS and lignin polymers are primarily comprise carbon chain and an aromatic ring side chain [26], and their degradations can be characterized by main-chain (C–C) or side-chain (benzene ring) oxidation, aromatic compound cleavage, and long-chain fatty acid degradation [35, 64, 65]. Previous studies found that the enzymes CYP4/6, monooxygenase, and aldehyde dehydrogenase were involved in PS metabolism of *G. mellonella* larvae [39], and reported CYP4/6 plays crucial role in the main-chain cleavage [73]. Another research reported that the PE plastic and beeswax degradation had similar metabolic approaches in *G. mellonella* due to the long-chain hydrocarbon as their major components [35]. Lac-, Mn-, and Lip-peroxidase can degrade lignin [67, 74]. In particular, some genes encoding LMCOs upregulated during plastic degradation [32], indicating a role in the cleavage of C–C bonds during the first step of PS and CS degradation. Representative enzymes for benzene ring opening, monooxygenase and peroxidase, and other key enzymes ADH, ALDH, and ACSL, were closely related to the process of long-chain fatty acid degradation [65, 75]. Our GC–MS and metabolomic analysis also detected some long-chain fatty acids, such as octadecenoic acid and hexadecenoic acid as the intermediates of PS/CS degradation in the larval gut or frass samples (Fig. 2E, F, J). Metatranscriptomics analysis, RT-PCR, GC–MS, and metabolomic analysis further suggested that the degradation pathways between PS and lignin are similar due to the analogous C–C backbone.

### Laccase enzyme activity and PS-/CS-degrading ability of Lac640

**Fig. 6:**
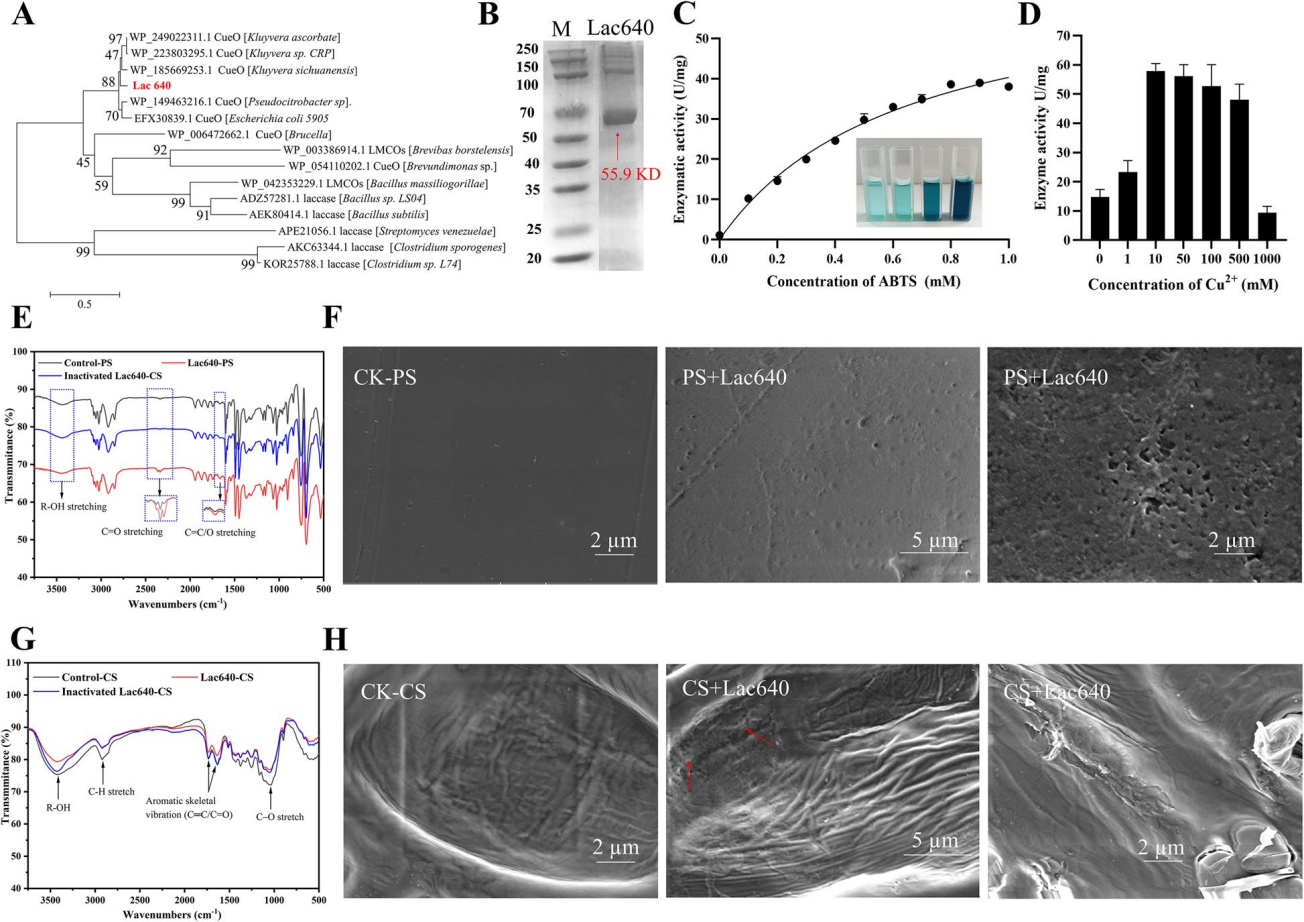
**A** Phylogenetic tree of Lac640 protein. **B** SDS-PAGE analysis of Lac640. Lane M, molecular size markers; lane Lac640, purified enzyme. **C** Laccase activity of Lac640 in the different concentrations of Cu^2+^. **D** Kinetic analysis of Lac640 at different concentrations of ABTS. **E**, **G** FTIR spectra for PS/CS samples treated with or without Lac640 enzyme; **F**, **H** The surface morphology of PS/CS treated with or without Lac640 enzyme

The metatranscriptomic data indicated that *lac640* encodes LMCOs, which belong to the AA1 family based on the CAZy database annotation. The protein Lac640 was highly upregulated in the PS- and CS-fed larvae groups (Fig. 5A), its corresponding bacterial source *Kluyvera* genus exhibited higher abundance in the PS- or CS-fed groups based on the Nr database annotation of the metatranscriptomic and 16S rRNA bacterial community (Additional file 1: S1, Table S9 & Fig. S10). These suggested that Lac640 could degrade both PS and CS. The phylogenetic tree showed that the homology of Lac640 with laccase derived from *Kluyvera ascorbata* was 86.37% (Fig. 6A). The protein Lac640 was successfully purified and the protein size was 55.9 kDa (Fig. 6B). To determine the optimal enzymatic rection for PS degradation, we optimized the highest laccase activity condition. The laccase activity of Lac640 increased from 8.7 to 37.9 U/mg in the presence of 10 mM copper sulfate (Fig. 6D), indicating that Cu^2+^ is a cofactor of Lac640. The *K*m and *V*max of Lac640 toward ABTS were 0.64 mM and 66.18 mM/min, respectively (Fig. 6C). Lac640 showed its highest enzyme activity at a temperature of 60 °C and pH of 5.0 (Additional file 1: S1, Fig. S12). To further determine whether the Lac640 enzyme can degrade PS and CS, FTIR and SEM analyses were conducted under optimal reaction conditions. The FTIR results of the Lac640-treated PS sample showed that the peak intensities of the hydrophobic group (R-OH: 3200–3600 cm^−1^) and C = C stretching (2500–1500 cm^−1^) were slightly different from those of the control PS and inactivated-Lac640 treated PS (Fig. 6E). The FTIR spectra of Lac640-treated CS sample showed that the band intensities were markedly decreased compared with those of the control CS and inactivated-Lac640 treated CS, i.e., at the peaks of 3500 cm^−1^ (-OH stretching) and 1800–1500 cm^−1^ (C = C: aromatic skeletal vibrations) (Fig. 6G). This suggested that the Lac640 enhanced the CS hydrophilicity and oxidizes its aromatic skeleton. Moreover, the SEM of Lac640-treated PS films showed a markedly porous morphology, whereas control PS films retained a smooth surface (Fig. 6F). Similarly, the CS sample treated with Lac640 was observed to have a deformed and porous structure, whereas the surface of control CS sample was mostly ordered (Fig. 6H).

Previous studies reported that LMCOs have laccase activity, and the addition of Cu^2+^ can enhance their activities [46], which is consistent with the Lac640 activity. However, the functional groups associated with hydrophobic (R-OH) and aromatic ring chain (C = C) were slightly changed in the Lac640-treated PS and CS samples. Additionally, a new peak at 2500–2250 cm^−1^ representing C = O was generated in the Lac640-treated PS samples (Fig. 6E–G). These lines of evidence indicated that Lac640 can oxidize PS and CS, which is consistent with previous research [76]. Several fungi or bacteria can degrade PE or PS plastic and lignin using their extracellular enzymes, such as Lac, Lip-, and Mn-peroxidase [28, 68]. In particular, various studies have focused on lignin degradation by laccase, it is a multicopper oxidase due to the copper ions [46, 77]. Although several studies have reported that laccase is upregulated during the plastic degradation in microorganisms [32, 47]. There is a lack of strong evidence confirming whether laccase can degrade PS plastics. Our study suggested that the enzyme Lac640 was capable of degrading PS and CS, which further supported the transcriptomic results and metabolic pathways, and provided a potential candidate enzyme for the future development of plastic-degrading bioproducts.

Microbial degradation of plastics is a promising treatment for the sustainable recycling of plastics. In recent years, efforts to find novel and highly efficient microorganisms or enzymes for plastic degradation have mainly focused on the marine environment and landfills [3, 47], but there is still a lack of highly efficient strain resources. Our study found key microbes and associated enzymes that can degrade both PS and CS in the larval gut, indicating the larvae used similar enzymes in the PS and CS degradation pathway due to their analogous C–C backbone. Previous studies reported that a novel PET hydrolase LCC was screened from a leaf-branch compost system [78]. Similarly, R*g*PETase was found in the natural rubber-degrading bacterium *Rhizobacter gummiphilus* isolated from a rhizosphere soil sample in a botanical garden [79]. Moreover, plastic-degrading enzymes, including Lac, Mn-, and Lip-peroxidase were observed in lignin-degrading fungi [7, 18, 68]. The LMCO-encoding gene *lac640* was found in our study, and proven to exert plastic- and lignin-degrading functions. These findings indicate that the gut or microbiota of lignin-degrading insects can be used as a source for screening potential plastic degrading candidates. The PVC-degrading bacterium *Klebsiella variicola* was also identified from the gut of insects (*Spodoptera frugiperda*) which is known to degrade natural polymer lignocellulose [80]. This study advances our understanding of the co-degradation mechanism of PS plastic and natural polymer CS, and lays foundation for mining novel PS-degrading enzymes from lignin-related environments. In addition, the novel PS- and CS-degrading enzyme Lac640 from unculturable microbes of insect’s gut, contributing to broaden the pool of available plastic-degrading enzymes.

## Conclusions

*T. molitor* larvae can utilize natural polymer lignocelluloses (CS) and synthetic plastics (PS). The ability of larvae to biodegrade PS and CS was confirmed by FTIR, TGA and GPC analysis. Some long-chain fatty acids were observed as the main byproducts in frass or gut samples from the PS- and CS-fed larvae by GC–MS and metabolomics analysis. The larvae formed a similar gut microbiota structure during adaptation to the PS and CS diets. The co-related bacteria including *Spiroplasma* sp., *Serratia* sp., *Staphylococcus* sp., *Rhodococcus* sp., *Bacillus* sp., and *Pseudomonas* sp. were observed in the gut microbiota of PS- and CS-fed larvae. The metatranscriptomic analysis suggested that the potential enzymes LMCOs, CYP6/4, AGMO, ADHs, CES1, Lip, and ACSL were involved in the digestion of PS and CS, indicating that PS and lignin degradation are controlled by similar enzymes. Furthermore, the RT-PCR results indicated that Lac640 is highly expressed in PS- and CS-fed larvae, and it has PS- and CS-degrading ability. This illustrates that plastic-degrading enzymes are strongly related to natural polymer-degrading enzymes. Therefore, the knowledge on the lignin metabolic pathway should be useful to identify effective enzymes for the degradation of plastics.

## Supplementary Information

**Additional file 1.** 

**Additional file 2.** 

**Additional file 3.** 

## Data Availability

The raw data on microbial diversity, metatranscriptomic sequences of *T. molitor*, and *lac640* have been submitted to NCBI, with accession numbers PRJNA857507, PRJNA858987, and OP020932, respectively.

## Abbreviations

CRs: Consumption rates
SRs: Survival rates
PRs: Pupation rates
PS: Polystyrene
CS: Corn straw
CB: Cabbage
DEGs: Differentially expressed genes
FDR: False discovery rate
LMCOs: Laccase-like multicopper oxidases (LMCOs)
PCoA: Principal coordinate analysis
PCA: Principal component Analysis
TGA: Thermogravimetric analysis
FTIR: Fourier transform infrared spectroscopy

## References

[1] Tieso, Global production volume of thermoplastics by type 2020–2050. 2021. https://www.statista.com/statistics/1192886/thermoplastics-production-volume-by-type-globally/. Accessed 12 Aug 2022.

[2] Sharma S, Basu S, Shetti NP, Nadagouda MN, Aminabhavi TM (2021). Microplastics in the environment: occurrence, perils, and eradication. Chem Eng J..

[3] Bahl S, Dolma J, Singh JJ, Sehgal S. Biodegradation of plastics: a state of the art review, materials today. Proceedings. 2021;39:31–34. 10.1016/j.matpr.2020.06.096.

[4] APaço A, Duarte K, da Costa JP, Santos PS, Pereira R, Pereira ME, Freitas AC, Duarte AC, Rocha-Santos TA. Biodegradation of polyethylene microplastics by the marine fungus Zalerion maritimum. Sci Total Environ. 2017;586:10–15. 10.1016/j.scitotenv.2017.02.017. 10.1016/j.scitotenv.2017.02.017 28199874

[5] Jadaun JS, Bansal S, Sonthalia A, Rai AK, Singh SP. Biodegradation of plastics for sustainable environment. Bioresource Technol. 2022;347:126697. 10.1016/j.biortech.2022.126697. 10.1016/j.biortech.2022.126697 35026422

[6] Yang J, Yang Y, Wu W-M, Zhao J, Jiang L (2014). Evidence of polyethylene biodegradation by Bacterial Strains from the guts of plastic-eating waxworms. Environ Sci Technol..

[7] Yang Y, Yang J, Wu WM, Zhao J, Song Y, Gao L, Yang R, Jiang L (2015). Biodegradation and mineralization of polystyrene by plastic-eating mealworms: part 2 role of gut microorganisms. Environ Sci Technol.

[8] Yang Y, Wang J, Xia M. Biodegradation and mineralization of polystyrene by plastic-eating superworms Zophobas atratus. Sci Total Environ. 2020;708:135233. 10.1016/j.scitotenv.2019.135233. 10.1016/j.scitotenv.2019.135233 31787276

[9] Brandon AM, Gao S-H, Tian R, Ning D, Yang S-S, Zhou J, Wu W-M, Criddle CS (2018). Biodegradation of polyethylene and plastic mixtures in mealworms (Larvae of Tenebrio molitor) and effects on the gut microbiome. Environ Sci Technol.

[10] Lou Y, Ekaterina P, Yang SS, Lu B, Liu B, Ren N, Corvini PF, Xing D. Biodegradation of polyethylene and polystyrene by greater wax moth larvae (Galleria mellonella L.) and the effect of co-diet supplementation on the core gut microbiome. Environ Sci Technol. 2020;54(5):2821–2831. 10.1021/acs.est.9b07044. 10.1021/acs.est.9b07044 32013402

[11] Peng BY, Li Y, Fan R, Chen Z, Chen J, Brandon AM, Criddle CS, Zhang Y, Wu WM. Biodegradation of low-density polyethylene and polystyrene in superworms, larvae of Zophobas atratus (Coleoptera: Tenebrionidae): broad and limited extent depolymerization. Environ Pollut. 2020;266:115206. 10.1016/j.envpol.2020.115206. 10.1016/j.envpol.2020.115206 32682160

[12] Peng BY, Chen Z, Chen J, Yu H, Zhou X, Criddle CS, Wu WM, Zhang Y. Biodegradation of Polyvinyl Chloride (PVC) in Tenebrio molitor (Coleoptera: Tenebrionidae) larvae. Environ Int. 2020;145:106106. 10.1016/j.envint.2020.106106. 10.1016/j.envint.2020.106106 32947161

[13] Song Y, Qiu R, Hu J, Li X, Zhang X, Chen Y, Wu WM, He D. Biodegradation and disintegration of expanded polystyrene by land snails Achatina fulica. Sci Total Environ. 2020;746:141289. 10.1016/j.scitotenv.2020.141289. 10.1016/j.scitotenv.2020.141289 32745868

[14] Lwanga EH, Thapa B, Yang X, Gertsen H, Salánki T, Geissen V, Garbeva P. Decay of low-density polyethylene by bacteria extracted from earthworm's guts: a potential for soil restoration. Sci Total Environ. 2018;624:753–757. 10.1016/j.scitotenv.2017.12.144. 10.1016/j.scitotenv.2017.12.144 29272844

[15] Xie S, Lan Y, Sun C, Shao Y (2019). Insect microbial symbionts as a novel source for biotechnology. World J Microbiol Biotechnol.

[16] G. Handique, A. Phukan, B. Bhattacharyya, A.A. Baruah, S.W. Rahman, R. Baruah, Characterization of cellulose degrading bacteria from the larval gut of the white grub beetle Lepidiota mansueta (Coleoptera: Scarabaeidae). Arch Insect Biochem Physiol. 2017;94(2). 10.1002/arch.21370. 10.1002/arch.21370 28094878

[17] Ceja-Navarro JA, Karaoz U, Bill M, Hao Z, White RA, Arellano A, Ramanculova L, Filley TR, Berry TD, Conrad ME, Blackwell M, Nicora CD, Kim YM, Reardon PN, Lipton MS, Adkins JN, Pett-Ridge J, Brodie EL (2019). Gut anatomical properties and microbial functional assembly promote lignocellulose deconstruction and colony subsistence of a wood-feeding beetle. Nat Microbiol.

[18] Tsegaye B, Balomajumder C, Roy P (2019). Isolation and characterization of novel lignolytic, cellulolytic, and hemicellulolytic bacteria from wood-feeding termite cryptotermes brevis. Int Microbiol.

[19] Ni J, Tokuda G. Lignocellulose-degrading enzymes from termites and their symbiotic microbiota. Biotechnol Adv. 2013;31(6):838–850. 10.1016/j.biotechadv.2013.04.005. 10.1016/j.biotechadv.2013.04.005 23623853

[20] Luo C, Li Y, Chen Y, Fu C, Long W, Xiao X, Liao H, Yang Y (2019). Bamboo lignocellulose degradation by gut symbiotic microbiota of the bamboo snout beetle Cyrtotrachelus buqueti. Biotechnol Biofuels.

[21] Finke M, Winn D (2004). Insects and related arthropods: a nutritional primer for rehabilitators. J Wildlife Rehabil..

[22] Paddock FB. The beemoth or waxworm, Texas agricultural experiment station. 1918.

[23] He L, Zhang Y, Ding MQ, Li MX, Ding J, Bai SW, Wu QL, Zhao L, Cao GL, Ren NQ, Yang SS. Sustainable strategy for lignocellulosic crop wastes reduction by Tenebrio molitor Linnaeus (mealworm) and potential use of mealworm frass as a fertilizer. J Clean Prod. 2021;325:129301. 10.1016/j.jclepro.2021.129301.

[24] Stotz HU, Kroymann J, Mitchell-Olds T. Plant-insect interactions. Curr Opin Plant Biol. 1999;2(4):268–272. 10.1016/S1369-5266(99)80048-X. 10.1016/S1369-5266(99)80048-X 10458997

[25] Ali SS, Elsamahy T, Al-Tohamy R, Zhu D, Mahmoud YA, Koutra E, Metwally MA, Kornaros M, Sun J. Plastic wastes biodegradation: mechanisms, challenges and future prospects. Sci Total Environ. 2021;780:146590. 10.1016/j.scitotenv.2021.146590. 10.1016/j.scitotenv.2021.146590 34030345

[26] Chen C-C, Dai L, Ma L, Guo R-T (2020). Enzymatic degradation of plant biomass and synthetic polymers. Nat Rev Chem.

[27] Jeyakumar D, Chirsteen J, Doble M. Synergistic effects of pretreatment and blending on fungi mediated biodegradation of polypropylenes. Bioresource Technol. 2013;148:78–85. 10.1016/j.biortech.2013.08.074. 10.1016/j.biortech.2013.08.074 24045194

[28] Mikulášová M, Košíková B, Alexy P, Kačík F, Urgelová E (2001). Effect of blending lignin biopolymer on the biodegradability of polyolefin plastics. World J Microbiol Biotechnol.

[29] Nakamiya K, Ooi T, Kinoshita S. Non-heme hydroquinone peroxidase from Azotobacter beijerinckii HM121. J Ferment Bioeng. 1997;84(1):14–21. 10.1016/S0922-338X(97)82780-8.

[30] Kavitha R, Bhuvaneswari V (2021). Assessment of polyethylene degradation by biosurfactant producing ligninolytic bacterium. Biodegradation.

[31] Nakamiya K, Sakasita G, Ooi T, Kinoshita S. Enzymatic degradation of polystyrene by hydroquinone peroxidase of Azotobacter beijerinckii HM121. J Ferment Bioeng. 1997;84(5):480–482. 10.1016/S0922-338X(97)82013-2.

[32] Zhang J, Gao D, Li Q, Zhao Y, Li L, Lin H, Bi Q, Zhao Y. Biodegradation of polyethylene microplastic particles by the fungus Aspergillus flavus from the guts of wax moth Galleria mellonella. Sci Total Environ. 2020;704:135931. 10.1016/j.scitotenv.2019.135931. 10.1016/j.scitotenv.2019.135931 31830656

[33] Inderthal H, Tai SL, Harrison ST. Non-hydrolyzable plastics – an interdisciplinary look at plastic bio-oxidation. Trends Biotechnol. 2021;39(1):12–23. 10.1016/j.tibtech.2020.05.004. 10.1016/j.tibtech.2020.05.004 32487438

[34] Peng B-Y, Sun Y, Xiao S, Chen J, Zhou X, Wu W-M, Zhang Y (2022). Influence of polymer size on polystyrene biodegradation in mealworms (Tenebrio molitor): responses of depolymerization pattern, gut microbiome, and metabolome to polymers with low to ultrahigh molecular weight. Environ Sci Technol..

[35] Kong HG, Kim HH, Chung JH, Jun J, Lee S, Kim HM, Jeon S, Park SG, Bhak J, Ryu CM. The galleria mellonella hologenome supports microbiota-independent metabolism of long-chain hydrocarbon beeswax. Cell Rep. 2019;26(9):2451–2464.e5. 10.1016/j.celrep.2019.02.018. 10.1016/j.celrep.2019.02.018 30811993

[36] Yang L, Gao J, Liu Y, Zhuang G, Peng X, Wu WM, Zhuang X. Biodegradation of expanded polystyrene and low-density polyethylene foams in larvae of Tenebrio molitor Linnaeus (Coleoptera: Tenebrionidae): broad versus limited extent depolymerization and microbe-dependence versus independence. Chemosphere. 2021;262:127818. 10.1016/j.chemosphere.2020.127818. 10.1016/j.chemosphere.2020.127818 32771707

[37] Yang SS, Kang JH, Xie TR, He L, Xing DF, Ren NQ, Ho SH, Wu WM. Generation of high-efficient biochar for dye adsorption using frass of yellow mealworms (larvae of Tenebrio molitor Linnaeus) fed with wheat straw for insect biomass production. J Clean Prod. 2019;227:33–47. 10.1016/j.jclepro.2019.04.005.

[38] Cui T, Yuan B, Guo H, Tian H, Wang W, Ma Y, Li C, Fei Q (2021). Enhanced lignin biodegradation by consortium of white rot fungi: microbial synergistic effects and product mapping. Biotechnol Biofuels.

[39] Wang S, Shi W, Huang Z, Zhou N, Xie Y, Tang Y, Hu F, Liu G, Zheng H. Complete digestion/biodegradation of polystyrene microplastics by greater wax moth (Galleria mellonella) larvae: Direct in vivo evidence, gut microbiota independence, and potential metabolic pathways. J Hazard Mat. 2022;423:127213. 10.1016/j.jhazmat.2021.127213. 10.1016/j.jhazmat.2021.127213 34844347

[40] Lou Y, Li Y, Lu B, Liu Q, Yang SS, Liu B, Ren N, Wu WM, Xing D. Response of the yellow mealworm (Tenebrio molitor) gut microbiome to diet shifts during polystyrene and polyethylene biodegradation. J Hazard Mat. 2021;416;126222. 10.1016/j.jhazmat.2021.126222. 10.1016/j.jhazmat.2021.126222 34492977

[41] Pei Y, Tao C, Ling Z, Yu Z, Ji J, Khan A, Mamtimin T, Liu P, Li X. Exploring novel Cr(VI) remediation genes for Cr(VI)-contaminated industrial wastewater treatment by comparative metatranscriptomics and metagenomics. Sci Total Environ. 2020;742:140435. 10.1016/j.scitotenv.2020.140435. 10.1016/j.scitotenv.2020.140435 32623159

[42] Robinson MD, McCarthy DJ, Smyth GK (2009). edgeR: a Bioconductor package for differential expression analysis of digital gene expression data. Bioinformatics.

[43] Benjamini Y, Hochberg Y. Controlling the False Discovery Rate: A Practical and Powerful Approach to Multiple Testing. J Royal Stat Soc B (Methodological). 1995; 57(1):289–300. 10.1111/j.2517-6161.1995.tb02031.x.

[44] Conesa A, Götz S, García-Gómez JM, Terol J, Talón M, Robles M (2005). Blast2GO: a universal tool for annotation, visualization and analysis in functional genomics research. Bioinformatics.

[45] Jang HA, Park KB, Kim BB, Ali Mohammadie Kojour M, Bae YM, Baliarsingh S, Lee YS, Han YS, Jo YH. Bacterial but not fungal challenge up-regulates the transcription of Coleoptericin genes in Tenebrio molitor. Entomol Res. 2020;50(9):440–449. 10.1111/1748-5967.12465.

[46] Zhang W, Wang W, Wang J, Shen G, Yuan Y, Yan L, Tang H, Wang W. Isolation and characterization of a novel laccase for lignin degradation, LacZ1. App Environ Microbiol. 2021;87(23):e0135521. 10.1128/aem.01355-21. 10.1128/AEM.01355-21 PMC8580002 34524901

[47] Kim HW, Jo JH, Kim YB, Le TK, Cho CW, Yun CH, Chi WS, Yeom SJ. Biodegradation of polystyrene by bacteria from the soil in common environments. J Hazard Mat. 2021;416:126239. 10.1016/j.jhazmat.2021.126239. 10.1016/j.jhazmat.2021.126239 34492990

[48] Yang SS, Zhang Y, Zhou HM, Ji XY, He L, Xing DF, Ren NQ, Ho SH, Wu WM. A novel clean production approach to utilize crop waste residues as co-diet for mealworm (Tenebrio molitor) biomass production with biochar as byproduct for heavy metal removal. Environ Poll. 2019;252:1142–53. 10.1016/j.envpol.2019.06.028. 10.1016/j.envpol.2019.06.028 31252112

[49] Peng BY, Su Y, Chen Z, Chen J, Zhou X, Benbow ME, Criddle CS, Wu WM, Zhang Y (2019). Biodegradation of Polystyrene by Dark ( Tenebrio obscurus) and Yellow ( Tenebrio molitor) Mealworms (Coleoptera: Tenebrionidae). Environ Sci Technol.

[50] Yang SS, Brandon AM, Flanagan JC, Yang J, Ning D, Cai SY, Fan HQ, Wang ZY, Ren J, Benbow E, Ren NQ, Waymouth RM, Zhou J, Criddle CS, Wu WM, Biodegradation of polystyrene wastes in yellow mealworms (larvae of Tenebrio molitor Linnaeus): factors affecting biodegradation rates and the ability of polystyrene-fed larvae to complete their life cycle. Chemosphere. 2018;191:979–89. 10.1016/j.chemosphere.2017.10.117. 10.1016/j.chemosphere.2017.10.117 29145143

[51] Brandon AM, Garcia AM, Khlystov NA, Wu W-M, Criddle CS (2021). Enhanced Bioavailability and Microbial Biodegradation of Polystyrene in an Enrichment Derived from the Gut Microbiome of Tenebrio molitor (Mealworm Larvae). Environ Sci Technol.

[52] Chen H, Liu J, Chang X, Chen D, Xue Y, Liu P, Lin H, Han S. A review on the pretreatment of lignocellulose for high-value chemicals. Fuel Proc Technol. 2017;160:196–206. 10.1016/j.fuproc.2016.12.007.

[53] Weng C, Peng X, Han Y (2021). Depolymerization and conversion of lignin to value-added bioproducts by microbial and enzymatic catalysis. Biotechnol Biofuels.

[54] Zhao L, Zhang J, Zhao D, Jia L, Qin B, Cao X, Zang L, Lu F, Liu F. Biological degradation of lignin: a critical review on progress and perspectives. Ind Crops Prod. 2022;188:115715. 10.1016/j.indcrop.2022.115715.

[55] Jönsson LJ, Martín C. Pretreatment of lignocellulose: formation of inhibitory by-products and strategies for minimizing their effects. Bioresource Technol. 2016;199:103–12. 10.1016/j.biortech.2015.10.009. 10.1016/j.biortech.2015.10.009 26482946

[56] Yang SS, Ding MQ, He L, Zhang CH, Li QX, Xing DF, Cao GL, Zhao L, Ding J, Ren NQ, Wu WM. Biodegradation of polypropylene by yellow mealworms (Tenebrio molitor) and superworms (Zophobas atratus) via gut-microbe-dependent depolymerization. Sci Total Environ. 2021;756:144087. 10.1016/j.scitotenv.2020.144087. 10.1016/j.scitotenv.2020.144087 33280873

[57] Woo S, Song I, Cha HJ (2020). Fast and Facile Biodegradation of Polystyrene by the Gut Microbial Flora of Plesiophthalmus davidis Larvae. Appl Environ Microbiol.

[58] Wang J, Liu X, Dai Y, Ren J, Li Y, Wang X, Zhang P, Peng C. Effects of co-loading of polyethylene microplastics and ciprofloxacin on the antibiotic degradation efficiency and microbial community structure in soil. Sci Total Environ. 2020;741:140463. 10.1016/j.scitotenv.2020.140463. 10.1016/j.scitotenv.2020.140463 32886986

[59] Badahit G, Singh A, Shrestha J, Chaudhary R, Rijal S (2018). Screening of Plastic Degrading Pseudomonas spp. From Soil, International Journal of Scientific and Engineering Research.

[60] Egwim E, Kabiru A, Tola A (2015). Partial characterization of lignin peroxidase expressed by bacterial and fungal isolates from termite gut, Biokemistri An International Journal of the Nigerian Society for. Exp Biol.

[61] Jiménez DJ, Korenblum E, van Elsas JD (2014). Novel multispecies microbial consortia involved in lignocellulose and 5-hydroxymethylfurfural bioconversion. Appl Microbiol Biotechnol.

[62] Zampolli J, Orro A, Manconi A, Ami D, Natalello A, Di Gennaro P (2021). Transcriptomic analysis of Rhodococcus opacus R7 grown on polyethylene by RNA-seq. Sci Rep.

[63] Sowmya HV, Krishnappa M, Thippeswamy B. Degradation of polyethylene by penicillium simplicissimum isolated from local dumpsite of Shivamogga district. Environ Dev Sustainabil. 2015;17(4):731–45. 10.1007/s10668-014-9571-4.

[64] Zhong Z, Nong W, Xie Y, Hui JH, Chu LM. Long-term effect of plastic feeding on growth and transcriptomic response of mealworms (Tenebrio molitor L.). Chemosphere. 2022;287:132063. 10.1016/j.chemosphere.2021.132063. 10.1016/j.chemosphere.2021.132063 34523442

[65] LeMoine CMR, Grove HC, Smith CM, Cassone BJ (2020). A Very Hungry Caterpillar: Polyethylene Metabolism and Lipid Homeostasis in Larvae of the Greater Wax Moth (Galleria mellonella). Environ Sci Technol.

[66] Bredon M, Dittmer J, Noël C, Moumen B, Bouchon D (2018). Lignocellulose degradation at the holobiont level: teamwork in a keystone soil invertebrate. Microbiome.

[67] Ni J, Tokuda G. Lignocellulose-degrading enzymes from termites and their symbiotic microbiota. Biotechnol Adv. 2013;31(6):838–50. 10.1016/j.biotechadv.2013.04.005. 10.1016/j.biotechadv.2013.04.005 23623853

[68] Zerva A, Siaperas R, Taxeidis G, Kyriakidi M, Vouyiouka S, Zervakis GI, Topakas E. Investigation of abortiporus biennis lignocellulolytic toolbox, and the role of laccases in polystyrene degradation. Chemosphere. 2023;312:137338. 10.1016/j.chemosphere.2022.137338. 10.1016/j.chemosphere.2022.137338 36423718

[69] Rashid GM, Taylor CR, Liu Y, Zhang X, Rea D, Fülöp V, Bugg TD. Identification of manganese superoxide dismutase from sphingobacterium sp. T2 as a novel bacterial enzyme for lignin oxidation. ACS Chem Biol. 2015;10(10):2286–94. 10.1021/acschembio.5b00298. 10.1021/acschembio.5b00298 26198187

[70] Raychoudhury R, Sen R, Cai Y, Sun Y, Lietze VU, Boucias DG, Scharf ME. Comparative metatranscriptomic signatures of wood and paper feeding in the gut of the termite Reticulitermes flavipes (Isoptera: Rhinotermitidae). 2013;22(2):155–71. 10.1111/imb.12011. 10.1111/imb.12011 23294456

[71] Kumar A, Naraian R. Chapter 6 - Differential Expression of the Microbial β-1,4-Xylanase, and β-1,4-Endoglucanase Genes, in: H.B. Singh, V.K. Gupta, S. Jogaiah (Eds.), New and future developments in microbial biotechnology and bioengineering, Amsterdam: Elsevier; 2019. p. 95–111. 10.1016/B978-0-444-63503-7.00006-1.

[72] Cong B, Wang N, Liu S, Liu F, Yin X, Shen J (2017). Isolation, characterization and transcriptome analysis of a novel Antarctic Aspergillus sydowii strain MS-19 as a potential lignocellulosic enzyme source. BMC Microbiol.

[73] Hou L, Majumder EL. Potential for and distribution of enzymatic biodegradation of polystyrene by environmental microorganisms. Materials (Basel, Switzerland). 2021;14(3). 10.3390/ma14030503. 10.3390/ma14030503 PMC7864516 33494256

[74] Tom LM, Aulitto M, Wu Y-W, Deng K, Gao Y, Xiao N, Rodriguez BG, Louime C, Northen TR, Eudes A, Mortimer JC, Adams PD, Scheller HV, Simmons BA, Ceja-Navarro JA, Singer SW (2022). Low-abundance populations distinguish microbiome performance in plant cell wall deconstruction. Microbiome.

[75] Chenprakhon P, Wongnate T, Chaiyen P. Monooxygenation of aromatic compounds by flavin-dependent monooxygenases. Protein Sci. 2019;28(1):8–29. 10.1002/pro.3525. 10.1002/pro.3525 PMC6295904 30311986

[76] Avanthi A, Banerjee R. A strategic laccase mediated lignin degradation of lignocellulosic feedstocks for ethanol production. Industr Crops Prod. 2016;92:174–85. 10.1016/j.indcrop.2016.08.009.

[77] Granja-Travez RS, Bugg TD. Characterization of multicopper oxidase CopA from pseudomonas putida KT2440 and pseudomonas fluorescens Pf-5: involvement in bacterial lignin oxidation. Arch Biochem Biophys. 2018;660:97–107. 10.1016/j.abb.2018.10.012. 10.1016/j.abb.2018.10.012 30347180

[78] Sulaiman S, Yamato S, Kanaya E, Kim JJ, Koga Y, Takano K, Kanaya S (2012). Isolation of a novel cutinase homolog with polyethylene terephthalate-degrading activity from leaf-branch compost by using a metagenomic approach. Appl Environ Microbiol.

[79] Sagong HY, Son HF, Seo H, Hong H, Lee D, Kim KJ. Implications for the PET decomposition mechanism through similarity and dissimilarity between PETases from Rhizobacter gummiphilus and Ideonella sakaiensis. J Hazard Mat. 2021;416:126075. 10.1016/j.jhazmat.2021.126075. 10.1016/j.jhazmat.2021.126075 34492896

[80] Zhang Z, Peng H, Yang D, Zhang G, Zhang J, Ju F (2022). Polyvinyl chloride degradation by a bacterium isolated from the gut of insect larvae. Nat Commun.

