# Supplementary material for: Gut microbiome of mealworms (Tenebrio molitor Larvae) show similar responses to polystyrene and corn straw diets
Source: Microbiome. 2023 May 5;11:98. doi: 10.1186/s40168-023-01550-w (PMC10161430; doi:10.1186/s40168-023-01550-w)
Supplement: Supplementary file 2 — Additional file 1. [file 40168_2023_1550_MOESM1_ESM.docx]

**Gut Microbiome of Mealworms (*Tenebrio molitor* Larvae) Show Similar Responses to Polystyrene and Corn Straw Diets**

Tursunay Mamtimin ^a^, Huawen Han ^a,b *^, Aman Khan ^a^, Pengya Feng ^a^, Qing Zhang ^a^, Xiaobiao Ma ^a^, Yitian Fang ^c^, Pu Liu ^a^, [Saurabh Kulshrestha](https://www.sciencedirect.com/science/article/pii/S0960852419316463#!) ^d^, Toshiro Shigaki ^e^, Xiangkai Li ^a, *^

^a^ Ministry of Education Key Laboratory of Cell Activities and Stress Adaptations, School of Life Science, Lanzhou University, Lanzhou, China

^b^ State Key Laboratory of Grassland Agro-ecosystems, Center for Grassland Microbiome, Lanzhou University, Lanzhou, China

^c^ State Key Laboratory of Microbial Metabolism, School of Life Sciences and Biotechnology, Shanghai Jiao Tong University, Shanghai, China

^d^ Faculty of Applied Sciences and Biotechnology, Shoolini University, Solan, India

^e^ Graduate School of Agricultural and Life Sciences, The University of Tokyo, Tokyo, Japan

* Corresponding Authors:

Prof. Xiangkai Li, [xkli@lzu.edu.cn](mailto:xkli@lzu.edu.cn), Tel. /fax: +86 931 8912562

Dr. Huawen Han, [hanhw@lzu.edu.cn](mailto:hhw@lzu.edu.cn), Tel. /fax: +86 931 8912562

**Supplementary Methods**

**M1. The Mass balance of PS and CS by larvae degradation**

To calculate the conversion rates of PS and CS，all frass samples of each feedstocks-fed larvae were collected at the end of 30 days.

**For PS mass balance:**

To estimate the PS removal (digested) rates were calculated the water-extracted fraction (*C*_w_), ethanol-extracted fraction (*C*_e_), and THF-extracted fraction (*C*_t_), based on the total mass of PS consumed, the mass of frass generated, and the residual PS content in the frass [1].

*C*_w_ = Mass of frass (g) − Water extracted dried residual frass (g)/ Mass of frass (g)ｘ100% (1)

*C*_e=_ Water extracted dried residual frass (g) − ethanol-extracted dried residual frass (g)/ Mass of frass (g) ｘ100% (2)

*C*_t=_ Ethanol-extracted dried residual frass (g) - THF-extracted dried residual frass (g) / Mass of frass (g)

ｘ100% (3)

PS removal (%)=(Total consumed PS (g) - Generated frass (g) ｘ*C*_t_ )/ total mass of PS consumed (g)

ｘ100% (4)

**For CS/CB mass balance calculate：**

To calculate the digested CS and CB we collected the frass samples and measured its lignocellulose component of CS and CB based on the previous study [2].

**Conversion rates %**=

Consumed feedstock (g) −Yield frass (g)/ Consumed feedstock (g)ｘ100% (1)

**Component consumed (g)**=

Total feedstock consumed (g)ｘlignocellulose component of feedstocks(CB,CS) (%) (2)

**Component digested (%)** =

Component consumed (g) − Component in frass (g)/ Component consumed (g)ｘ100% (3)

**M2. Gel permeation chromatography**

Gel permeation chromatography (GPC) of PS foam and frass form PS-fed larvae were measured. The PS foam (0.05g) and frass samples (0.5g) were dried at ambient temperature, then extracted 2h with 20 ml of tetrahydrofuran (THF; 120 mL, purity ≥99.9%). The THF solution was mixed with samples and heating (50 ℃) for 2h, then filtered by 0.22um Nylon membrane filter. The filtered solution was concentrated to 5 mL/mL in volume, the extract (50 μL in volume) was injected into the GPC analyzer, with a flow rate of 0.8 mL/ min [4] .

**M3. Lignocellulose component measuring methods**

Lignin, cellulose and hemicellulose contents of the CS, CB and their frass samples from the were measured by using van soest method (ANKOM220 Fiber analyzer) [3]. Equations used for the calculation are described as follows:

Hemicellulose (%) =NDF% − ADF%

Lignin (%) =ADL%

Cellulose (%) =ADF% − Residue after 72%H_2_ SO_4_

**M4. Gas chromatography-mass spectrometry (GC-MS)**

The control PS/CS samples, intestine and frass samples were pretreated to prior to GC-MS analysis. Briefly, the 0.1 g frass sample and guts of PS-fed larvae (n=5) were extracted with 10 mL of THF/THF: methanol (2:1), respectively. The samples were gently heated for 2 h and immersed for 16 h, and the solvents were evaporated, then residual polymers were redissolved in 100% hexane for the GC-MS analysis. For the pretreatment CS-fed samples, 0.1 g frass and guts of CS-fed larvae (n=5) were placed in glass tubes with 2 mL of tetramethylammonium hydroxide (TMAH) (25 wt % TMAH in methanol). The methanol evaporated under a stream of nitrogen gas, then samples transferred high temperature resistant ceramic crucible and put it in an oven at 250℃ for 30 min. The 1ml of ethyl acetate was added after the samples cooled and mixed with residual samples. The liquid was filtered with a 0.22 µm nylon membrane to GC-MS analysis. The PS/CS samples underwent the same pretreatment and were treated as the control groups. The GC-MS analysis was performed with a Thermo Trace 1300 GC coupled to a flame ionization detector and a Thermo ISQ mass spectrometer (Thermo, San Jose, California, U.S.A.), equipped with an TG-5 MS (30 m × 0.25 mm × 0.25 μm, Thermo) column and helium as the carrier gas. The oven temperature programming started at c [5]. The compounds were identified based on the NIST11 database.

### M5. Comparative metabolomic analysis

**Sample preparation method:** The extracted gut tissue of mealworms was immediately immersed into pre-cooled methanol/acetonitrile/water solution (2:2:1, v/v) and vortex mixing. Then, ultrasonic at low temperature for 30 min, and placed at -20℃ for 10 min, centrifuge at 4℃ for 20 min at 14000 g, supernatant was vacuum dried. 100 μL acetonitrile water solution (acetonitrile :Water =1:1, v/v) was added during mass spectrometry, and vortex, centrifuged at 4℃ for 15 min, at14000 g, the final supernatant was used for sample analysis [6].

**Chromatographic analysis:** The samples were separated by Agilent 1290 Infinity LC ultra-high performance Liquid chromatography (UHPLC) HILIC column. Column temperature 25℃; Flow rate 0.5 mL /min; [Injection Volume](javascript:;) 2 μL; The samples were placed in an automatic injector at 4℃ during the whole analysis. In order to avoid the influence caused by the fluctuation of the instrument detection signal, the random sequence of samples is used for continuous analysis.

**Mass spectrometry identification:** Positive and negative ion modes of electrospray ionization (ESI) were used for detection. The samples were separated by UHPLC and analyzed by Triple TOF 6600 mass spectrometers (AB SCIEX). After the samples were separated by Agilent 1290 Infinity LC ultra-high performance liquid chromatography (UHPLC), mass spectrometry was performed by Triple TOF 6600 mass spectrometer (AB SCIEX), and positive and negative ion modes of electrospray ionization (ESI) were used for detection [6].

**M6. RNA extraction and Illumina sequencing**

Fresh gut samples of larvae were collected, and total RNA was extracted using RNeasy PowerMicrobiome Kit (QIAGEN, GmbH, 26000-50) according to the manufacturer’s instruction. RNA quality and quantity were assessed via gel electrophoresis (1% agarose gels), a Nanodrop spectrophotometer (Agilent Alto, CA, USA) and with BioAnalyzer 2100 (Agilent Technologies, Santa Clara, CA). Total RNA samples were treated with the Ribo-Zero rRNA Removal kits (Epicentre) to remove environmental rRNA and using the TruSeq™ RNA Sample Prep Kit to converted to construction cDNA subsequent library [7]. Bridge PCR was performed before RNA was sequenced on an Illumina Hiseq (HiSeq 3000/4000 SBS Kits) X Ten platform (Majorbio).

**M7. Assembly and annotation of the meta-transcriptome**

Raw reads from the transcriptomes were preprocessed via quality trimming by Seqprep (https://github.com/jstjohn/SeqPrep) and Sickle (https://github.com/najoshi/sickle). Raw reads less than 50 bp were removed and trimmed to a minimum quality score of 20. Raw reads were filtered by SortMeRN (http://bioinfo.lifl.fr/ RNA/sortmerna/) to remove rRNA sequences [8]. High quality reads from each library were assembled using Trinity (http://trinityrnaseq.github.io/,version: trinityrnaseq-r2013-02-25) with minimum contig length of 300bp. ORF prediction was performed for all assembled transcripts using TransGeneScan software (<http://sourceforge.net/projects/transgenescan/>). Next, the all transcripts were combined and clustered into unique classes with CD-HIT (parameters were set at 95% identity, 90% coverage) to construct the non-redundant gene set. The gene expression is calculated using RSEM (RNA-Seq by Expectation Maximization; <http://deweylab.biostat.wisc.edu/rsem/>). For the functional annotations, the BLASTP (http://blast.ncbi.nlm.nih.gov/Blast.cgi) search (E-value ≤ 1e^-5^) of each unigene was conducted against the NCBI-nr database, Non-supervised Orthologous Groups (eggNOG, http://eggnog.embl.de), Gene Ontology (GO, http://www.geneontology.org) and Kyoto Encyclopedia of Genes and Genomes (KEGG, http://www.genome.jp/kegg/) databases to predict gene functions and metabolic pathways. CAZymes were identified using the Carbohydrate Active enZymes (CAZy) database (<http://www.cazy.org/>).

**M8. Expression and purification of protein Lac640**

The Lac640 gene was amplified with PrimeSTAR HS DNA Polymerase (TaKaRa), and subcloned into EcoR1 and Xho1 restriction enzymes sites before inserting them into the plasmid pET28a. Then, the reconstructed plasmids pET28a-lac640 were transformed into *E. coli* Rosetta (DE3) for protein expression. The *E. coli* (lac640) cells were grown to an OD_600_ of 0.6 at 37 ℃ in Luria-Bertani (LB) medium, a final concentration of 0.5 mM isopropyl β-D-1-thiogalactopyranoside (IPTG) was added, and cells were further cultivated at 16 ℃ for 16 h. The cells were harvested by centrifugation (8000 rpm/10 min), resuspended in binding buffer (20 mM Tris-HCl pH 8.0, 300 mM NaCl, 10 mM imidazole) and disrupted by ultrasonic cell disruptor followed centrifugation at the 12000 rpm/30 min to remove cell debris [9]. Next, the Lac640 protein with an N-terminal His tag was purified by Ni-chelating affinity chromatography, the bound protein was eluted with 300 mM imidazole from the Ni column. The purity of protein Lac640 was detected by using SDS-PAGE assay.

**Reference**

1. Peng B-Y, Sun Y, Xiao S, Chen J, Zhou X, Wu W-M, Zhang Y: **Influence of Polymer Size on Polystyrene Biodegradation in Mealworms (Tenebrio molitor): Responses of Depolymerization Pattern, Gut Microbiome, and Metabolome to Polymers with Low to Ultrahigh Molecular Weight**. *Environmental Science & Technology* 2022, **56**(23):17310-17320.

2. Yang S-S, Chen Y-d, Zhang Y, Zhou H-M, Ji X-Y, He L, Xing D-F, Ren N-Q, Ho S-H, Wu W-M: **A novel clean production approach to utilize crop waste residues as co-diet for mealworm (Tenebrio molitor) biomass production with biochar as byproduct for heavy metal removal**. *Environmental Pollution* 2019, **252**:1142-1153.

3. Yang S-S, Chen Y-d, Kang J-H, Xie T-R, He L, Xing D-F, Ren N-Q, Ho S-H, Wu W-M: **Generation of high-efficient biochar for dye adsorption using frass of yellow mealworms (larvae of Tenebrio molitor Linnaeus) fed with wheat straw for insect biomass production**. *Journal of Cleaner Production* 2019, **227**:33-47.

4. Yang L, Gao J, Liu Y, Zhuang G, Peng X, Wu W-M, Zhuang X: **Biodegradation of expanded polystyrene and low-density polyethylene foams in larvae of Tenebrio molitor Linnaeus (Coleoptera: Tenebrionidae): Broad versus limited extent depolymerization and microbe-dependence versus independence**. *Chemosphere* 2021, **262**:127818.

5. Jin W, Shen D, Liu Q, Xiao R: **Evaluation of the co-pyrolysis of lignin with plastic polymers by TG-FTIR and Py-GC/MS**. *Polymer Degradation and Stability* 2016, **133**:65-74.

6. León Z, García-Cañaveras JC, Donato MT, Lahoz A: **Mammalian cell metabolomics: Experimental design and sample preparation**. 2013, **34**(19):2762-2775.

7. Pei Y, Tao C, Ling Z, Yu Z, Ji J, Khan A, Mamtimin T, Liu P, Li X: **Exploring novel Cr(VI) remediation genes for Cr(VI)-contaminated industrial wastewater treatment by comparative metatranscriptomics and metagenomics**. *The Science of the total environment* 2020, **742**:140435.

8. Yu Z, Pei Y, Zhao S, Kakade A, Khan A, Sharma M, Zain H, Feng P, Ji J, Zhou T *et al*: **Metatranscriptomic analysis reveals active microbes and genes responded to short-term Cr(VI) stress**. *Ecotoxicology (London, England)* 2021, **30**(8):1527-1537.

9. Zhang W, Wang W, Wang J, Shen G, Yuan Y, Yan L, Tang H, Wang W: **Isolation and Characterization of a Novel Laccase for Lignin Degradation, LacZ1**. *Applied and environmental microbiology* 2021, **87**(23):e0135521.

**Supplementary data**

**Table S1.** Characteristics the three feedstocks used in this study.

| **Feedstock** | **Cellulose (dw%)** | **Hemicellulose (dw%)** | **Lignin (dw%)** | **N (%)** | **C (%)** | **C/N** |
| --- | --- | --- | --- | --- | --- | --- |
| CB | 35.4±2.9 | 8.3±1.3 | 2.5±1.2 | 5.82 | 41.22 | 7.08 |
| CS | 40.8±2.4 | 27.8±1.8 | 10.8±3.0 | 0.55 | 43.57 | 79.43 |
| Feedstock | Mw | Mn |  |  |  |  |
| PS | 120,000 Da | 73,178 Da |  |  |  |  |

**Table S2.** Information on primers of target and housekeeping genes for RT-PCR.

| Gene Number | Gene ID | Primer Fwd. (5'-3') | Primer Rev (5'-3') |
| --- | --- | --- | --- |
| Controls | Tml27a (60S ribosomal protein 27a) | TCATCCTGAAGGCAAAGCTCCAGT | AGGTTGGTTAGGCAGGCACCTTTA |
| Cytochrome P450 family 6 [EC:1.14.-.-] | comp4515_c0_seq1.1.419.minus.CS | GTAGTTCGACCATGGCGTTT | CAAAAACGGAAGCACTGGAT |
| Cytochrome P450 family 4 [EC:1.14.-.-] | comp11828_c0_seq1.1.348.minus.CS | TTGGGGTCGCTCATACTACC | CAAACCAGTTCCCAACCACT |
| Aldehyde dehydrogenase family [EC:1.2.1.3] | comp17350_c0_seq1.15.806.minus.CS | AAGTCGGGGATCCTTTTGAC | CGGTGGGCTGTACGAAGTAT |
| Alcohol dehydrogenase GroES-like domain [EC:3.1.2.12] | comp5385_c0_seq1.1.548.minus.PS | GCCTGAACTTCCAGGACTTG | AGCAGCGTCTAGATGGGAAA |
| Superoxide dismutase, Fe-Mn family [EC:1.15.1.1] | comp34094_c0_seq1.52.356.minus.PS | ACGGTGAGGAACTCTCTGTTATG | CTTGGTCCCAGTTTACTGTATGC |
| Long-chain-fatty-acid--CoA ligase ACSBG [EC:6.2.1.3] | comp12941_c0_seq1.1.766.minus.PS | GACCTCCGAAGCATATCCAA | GCGATGAACCATTCTGGACT |
| Cu-oxidase [EC 1.10.3.-] | comp12658_c0_seq1.1.371.minus.PS | GGATTGGGTCGCTTTAAACA | AAATCACTACCGTCGCTGCT |
| Laccase-like multicopper oxidase [EC 1.10.3.-] | comp16162_c0_seq1.1.802.minus.CK | CGCCTAAAACCCGACATAAA | TTATGGGTGCACATGCAGTT |
| Feruloyl esterase [EC 3.1.1.73] | comp13789_c0_seq1.1.1217.minus.PS | TCCAAAACACCAGAGATGAGTCT | TGTAGTTCTGGAAGCCTGAAGAG |
| Carboxyl esterase [EC 3.1.1.3] | comp17495_c0_seq1.1.744.minus.CS | AACGATGTAGTCCTGGTCACAGT | AAAGAACCGCTTTCTAGGATGAC |
| Acetyl xylan esterase [EC 3.1.1.72] | comp14315_c1_seq1.1.1066.minus.CK | ATTACCACATTTGCAAACATTCC | TATCGGACAGTCTTGGTTTGACT |
| Peroxidase [EC:1.11.1.7] | comp11737_c0_seq1.1.365.minus.CS | ACAATACAAGGACGACGAAAAGA | ACCAGCTGAAGAGAATCAATGAC |
| Alkylglycerol monooxygenase [EC:1.14.16.5] | comp16428_c0_seq1.1.933.minus.CS | AATTTTGGATACACACCAAAACG | CTGTAAATGGAGGATGTTGAAGG |
| Endo-beta-1,3(4)-glucanase [EC 3.2.1.6] | comp12927_c0_seq1.1.611.minus.CS | CTTCAGGGTATGGAGATGAGTTG | TCTTGATGGTTGTGATGTACTCG |
| Acyl-CoA oxidase [EC:1.3.3.6] | comp12231_c0_seq1.1.455.minus.CS | CGCCATATTAACGAAAGAATGAG | TCTTAGCAAATCGCCTGTTAGTC |
| Laccase-like Multicopper oxidase CueO  [EC 1.10.3.-] | Feces_k97_48640_gene_4_1 | CGTACCGGTCATCATCCAGG | GCTGGCAATGACATACAGCG |
| Lipase [EC 3.1.1.-] | comp15334_c0_seq1.1.1094.minus.CK | CCTCCATGGACATCAGAACA | GGTCGATTCCAGAAGCAGAA |

| Sample  name | Feed mass, g | Consumption rates, % | Generated frass mass, g | Conversion rates,  % | Lignocellulose content in feedstocks, % | | | Component consumed, g | Component in frass, g | | Component Digested, g | | Component Reduction  (Digestion rate),  % | | Average mealworm weight, mg | | |
| --- | --- | --- | --- | --- | --- | --- | --- | --- | --- | --- | --- | --- | --- | --- | --- | --- | --- |
|  |  |  |  |  |  |  |  |  |  |  |  |  |  |  | **Initial, mg** | **End, mg** | **Changes, %** |
| CB | 24.0$\pm$0.05 | 100% (24g) | 12.5$\pm$ 0.7 | 48.0$\pm$2.5 | Cellulose | 35.4$\pm$2.9 | | 8.5$\pm$0.7 | 4.0$\pm$0.5 | | 4.5$\pm$0.5 | | 52.9$\pm$1.4 | | 85.4$\pm$5.0 | 105.3$\pm$4.3 | 23.3$\pm$2.5 |
|  |  |  |  |  | Hemicellulose | 8.3$\pm$1.3 | | 2.0$\pm$0.3 | 1.2$\pm$0.4 | | 0.8$\pm$0.2 | | 40.0$\pm$2.2 | |  |  |  |
|  |  |  |  |  | Lignin | 2.5$\pm$1.0 | | 0.6$\pm$0.2 | 0.25$\pm$0.05 | | 0.34$\pm$0.1 | | 55.7$\pm$3.2 | |  |  |  |
| CS | 2.0$\pm$0.02 | 52% (1.1g) | 0.8$\pm$ 0.2 | 27.3$\pm$1.5 | Cellulose | 40.8$\pm$2.4 | | 0.45$\pm$ 0.03 | 0.25$\pm$0.03 | | 0.2$\pm$0.06 | | 44.4$\pm$5.3 | | 85.7$\pm$4.8 | 77.5$\pm$5.0 | -9.6$\pm$1.6 |
|  |  |  |  |  | Hemicellulose | 27.8$\pm$1.8 | | 0.31$\pm$0.03 | 0.22$\pm$0.04 | | 0.08$\pm$0.02 | | 26.6$\pm$5.1 |  | |  |  |
|  |  |  |  |  | Lignin | 10.8$\pm$3.0 | | 0.12$\pm$0.03 | 0.08$\pm$0.01 | | 0.04$\pm$0.01 | | 33.3$\pm$4.2 |  |  |  |  |
| PS | 2.0$\pm$ 0.02 | 32.5% (0.67g) | 0.54$\pm$0.1 | 19.4$\pm$2.0 | **Water-extracted fraction (Ce, %)** | | **Ethanol-extracted fraction (Ce, %)** | | | **THF-extracted fraction (Ct, %)** | | **PS removal rates (Digestion rate), (%)** | | 86.3$\pm$2.1 | | 70.8$\pm$2.5 | -18.6$\pm$1.0 |
|  |  |  |  |  | 18.48$\pm$1.4 | | 2.06$\pm$0.2 | | | 45.2$\pm$3.5 | | 63.49$\pm$3.4 | |  |  |  |  |

**Table S3.** The mass balance of three feedstocks (CB, CS and PS) by mealworms over a 30-day rearing period.

**Table S4.** The Mw, Mn, and Mz changes of PS extracted from frass versus feedstock PS foam.

| Samples | Mw (Da) | Mn (Da) | Mz (Da) | Mean Diff. Mw (Da) change in Mw (%) | Mean Diff. Mn (Da) change in Mn (%) | Mean Diff. Mz (Da) change in Mz (%) |
| --- | --- | --- | --- | --- | --- | --- |
| PS | 120,000$\pm$1383.6 | 73,178$\pm$516.1 | 150,774$\pm$4365.7 |  |  |  |
| PS-frass | 94,466$\pm$3644.5 | 59,151$\pm$3233.5 | 101,197$\pm$4935.3 | 25089$\pm$2276  20.9$\pm$1.9 % | 14027$\pm$3605  19.2$\pm$4.9 % | 49576$\pm$4219  32.8$\pm$2.8 % |
| PS-frass+An | 113,490$\pm$1396.1 | 67,786$\pm$2085.3 | 142,435$\pm$2247.5 | 6065$\pm$2143  5.0 $\pm$1.8 % | 5392 $\pm$ 2327  7.3 $\pm$ 3.1 % | 8339$\pm$5005  5.5$\pm$3.3 % |

An: Antibiotics

**Table S6.** Biodiversity index of total microbiota based on 16S rRNA sequencing.

| **Category** | **CK (CB)** | **PS** | **CS** |
| --- | --- | --- | --- |
| Sequences | 176,139 | 234,681 | 258,254 |
| average length | 428.24 | 428.11 | 428.28 |
| OTU | 96 | 131 | 58 |
| Shannon | 1.44 | 0.79 | 0.95 |
| Simpson | 0.34 | 0.66 | 0.57 |
| Ace | 51.93 | 66.6 | 40.6 |
| Chao | 50.25 | 58.17 | 36.91 |
| Coverage | 0.99 | 0.99 | 0.99 |

**Table S7.** The basic information of Meta-transcriptomic sequencing data.

| **Category** | **CK (CB) bp** | **PS bp** | **CS bp** |
| --- | --- | --- | --- |
| Reads from raw data | 88,176,838 | 90,197,992 | 90,039,432 |
| Clean reads | 87,468,710 | 89,290,414 | 89,150,052 |
| Non-rRNA reads | 15,414,648 | 14,032,970 | 19,149,682 |
| Contigs | 28,413 | 31,636 | 46,494 |
| N50 (bp) | 1138 | 1148 | 1421 |
| N90 (bp) | 399 | 392 | 423 |
| ORFs | 17,184 | 17,651 | 26,332 |
| Average length of ORFs (bp) | 820.81 | 835.72 | 939.13 |

**Table S5.** Identified compounds in the extract of the gut and Frass sample from biodegradation of polystyrene (PS) and Corn strow (CS) from *Tenebrio molitor*.

|  | **F-PS** | **G-PS** | **F-CS** | **G-CS** |
| --- | --- | --- | --- | --- |
| 1 | Benzene, 1,3-dimethyl (C_8_H_10_) | Benzene, 1,3-dimethyl- (C_8_H_10_) | Benzene, 1,3,5-tris(1-methylethyl) (C_15_H_24_) | Benzenepropanoic acid (C_18_H_28_O_3_) |
| 2 | Benzenepropanoic acid (C_18_H_28_O_3_) | Benzenepropanoic acid (C_18_H_28_O_3_) | Benzenepropanoic acid, octadecyl ester (C_35_H_62_O_3_) | Phenol, 2,4-bis(1,1-dimethylethyl)- (C_14_H_22_O) |
| 3 | Toluene (C_7_H_8_) | Furan, tetrahydro-2-methyl (C_5_H_10_O) | Eicosane, 2-methyl (C_21_H_44_) | Eicosane, 2-methyl (C_21_H_44_) |
| 4 | 3-Hydroxypropyl palmitate (C_22_H_46_O_3_) | 3-Hydroxypropyl palmitate (C_22_H_46_O_3_) | 2,4-Di-tert-butylphenol (C_14_H_22_O) | Octadecane, 3-ethyl-5-(2-ethylbutyl) (C_26_H_54_) |
| 5 | 2,4-Di-tert-butylphenol (C_14_H_22_O) | Ethyl iso-allocholate (C_26_H_44_O_5_) | Ethyl iso-allocholate (C_26_H_44_O_5_) | Ethyl iso-allocholate (C_26_H_44_O_5_) |
| 6 | Ethyl iso-allocholate (C_26_H_44_O_5_) | 2-Methoxytetrahydrofuran (C_5_H_10_O_2_) | Tetratetracontane (C_44_H_90_) | Tetratetracontane (C_44_H_90_) |
| 7 | 4-Ethylbenzoic acid, 6-ethyl-3-octyl ester (C_19_H_30_O_2_) | 2-Hydroxychalcone (C_15_H_12_O_2_) | Heptacosane (C_27_H_56_) | Phenol, 2,2‘-methylenebis[6-(1,1-dimethylethyl)-4-methyl- (C_23_H_32_O_2_) |
| 8 | 2-Methoxytetrahydrofuran (C_5_H_10_O_2_) | 4'-Hydroxychalcone (C_15_H_12_O_2_) | Cyclohexane 1,3,5-trimethyl-2-octadecyl (C_27_H_54_) | Cyclohexane 1,3,5-trimethyl-2-octadecyl (C_27_H_54_) |
| 9 | 2-Hydroxychalcone (C_15_H_12_O_2_) | Hexadecane (C_16_H_34_) | Hexadecanoic acid, octadecyl ester (C_34_H_68_O_2_) | 2-Hydroxychalcone (C_15_H_12_O_2_) |
| 10 | 4'-Hydroxychalcone (C_15_H_12_O_2_) | Octadecane, 3-ethyl-5-(2-ethylbutyl) (C_26_H_54_) | Hexadecanoic acid, methyl ester (C_17_H_34_O_2_) | Hexadecanoic acid, methyl ester (C_17_H_34_O_2_) |
| 11 | Cyclohexene (C_10_H_16_) | Propanoic acid, 2-(3-acetoxy-4,4,14-trimethylandrost-8-en-17-yl) (C_27_H_42_O_4_) | Propanoic acid, 2-(3-acetoxy-4,4,14-trimethylandrost-8-en-17-yl) (C_27_H_42_O_4_) | Propanoic acid, 2-(3-acetoxy-4,4,14-trimethylandrost-8-en-17-yl) (C_27_H_42_O_4_) |
| 12 | Tetradecane (C_14_H_30_) | Hexadecanoic acid, ethyl ester (C_18_H_36_O_2_) | 9,12-Octadecadienoic acid (C_19_H_34_O_2_) | Hexadecanoic acid, 14-methyl, (C_18_H_36_O_2_) |
| 13 | 11,14-Eicosadienoic acid (C_21_H_38_O_2_) | Octadecanoic acid, 2-propenyl ester (C_21_H_40_O_2_) | Decanoic acid, 9a-diyl ester (C_40_H_64_O_8_) | Dodecanoic acid, (C_32_H_48_O_6_) |
| 14 | Undecanoic acid (C_11_H_22_O_2_) | Oleic acid, 3-(octadecyloxy) propyl ester (C_39_H_76_O_3_) | Tetradecanoic acid, Methyl tetradecanoate (C_15_H_30_O_2_) | Tetradecanoic acid, Methyl tetradecanoate (C_15_H_30_O_2_) |
| 15 | 9,12-Octadecadienoic acid (C_20_H_36_O_2_) | 9,12-Octadecadienoic acid, ethyl ester (C_20_H_36_O_2_) | 9-Octadecenoic acid, methyl ester (C_19_H_36_O_2_) | 9-Octadecanoic acid, methyl ester (C_19_H_38_O_2_) |
| 16 | 3,6-Octadecadiynoic acid (C_19_H_30_O_2_) | Undecanoic acid (C_11_H_22_O_2_) | D- (+)-Galactose (C_16_H_21_NO_10_) | Acetic acid, 10-ylmethyl ester (C_25_H_39_NO_5_) |
| 17 | Butanoic acid, 4-hydroxy (C_4_H_8_O_3_) | Acetic acid, pentyl ester (C_7_H_14_O_2_) |  |  |
|  |  | Butanoic acid, 4-hydroxy (C_4_H_8_O_3_) |  |  |

**Table S8.** Activity of hydrolytic enzymes related to plastic and lignocellulose degradation of PS/CS fed mealworm.

|  | **Gene ID** | **KEEG/CAZy annotation** | **Identity** | **NCBI Definition** |
| --- | --- | --- | --- | --- |
| 1 | comp15805_c0_seq3.1.760.minus.CK_1 | Chitinase [EC:3.2.1.14] | 40.07% | [chitinase [*Gregarina niphandrodes*]](https://blast.ncbi.nlm.nih.gov/Blast.cgi) |
| 2 | comp8347_c0_seq1.1.1398.minus.CK_1 | Catalase [EC:1.11.1.6] | 100% | [catalase [*Hafnia alvei*]](https://blast.ncbi.nlm.nih.gov/Blast.cgi) |
| 3 | comp26924_c0_seq1.1.288.minus.CK_1 | Catalase-peroxidase [EC:1.11.1.21] | 100% | catalase-peroxidase [[*Enterobacteriaceae*](https://blast.ncbi.nlm.nih.gov/Blast.cgi#alnHdr_WP_106929385)] |
| 4 | comp14052_c0_seq2.1.304.minus.CK_1 | Alkyl hydroperoxide reductase subunit F [EC:1.6.4.-] | 94.00% | [alkyl hydroperoxide reductase [*S.enterica*]](https://blast.ncbi.nlm.nih.gov/Blast.cgi) |
| 6 | comp8716_c0_seq1.1.612.minus.CS_1 | Sulfur dioxygenase [EC:1.13.11.18] | 82.21% | [persulfide dioxygenase ETHE1, mitochondrial [](https://blast.ncbi.nlm.nih.gov/Blast.cgi)*T.* [*castaneum*]](https://blast.ncbi.nlm.nih.gov/Blast.cgi) |
| 7 | comp34094_c0_seq1.52.356.minus.PS_1 | Superoxide dismutase, Fe-Mn family [EC:1.15.1.1] | 100% | [superoxide dismutase [Mn] [*Bacillus cereus*]](https://blast.ncbi.nlm.nih.gov/Blast.cgi) |
| 8 | comp22182_c0_seq1.67.551.minus.CK_1 | Putative oxidoreductase | 34.67% | Putative oxidoreductase CatD [*Bacillus subtilis subsp*.] |
| 9 | comp21565_c0_seq1.1.367.minus.CK_1 | D-lactate dehydrogenase [EC:1.1.1.28] | 100% | [2-hydroxyacid dehydrogenase [*Hafnia alvei*]](https://blast.ncbi.nlm.nih.gov/Blast.cgi) |
| 10 | comp11737_c0_seq1.1.365.minus.CS_1 | Peroxidase [EC:1.11.1.7] | 91.74% | [peroxiredoxin-6 [*Tribolium castaneum*]](https://blast.ncbi.nlm.nih.gov/Blast.cgi) |
| 11 | comp7761_c0_seq1.1.339.minus.CS_1 | Pyruvate carboxylase [EC:6.4.1.1] | 89.81% | [pyruvate carboxylase [*T. castaneum*]](https://blast.ncbi.nlm.nih.gov/Blast.cgi) |
| 12 | comp22669_c0_seq1.1.360.minus.CK_1 | Acyloxyacyl hydrolase [EC:3.1.1.77] | 75.63% | [acyloxyacyl hydrolase [*Tribolium castaneum*]](https://blast.ncbi.nlm.nih.gov/Blast.cgi) |
| 13 | comp16428_c0_seq1.1.933.minus.CS_1 | Alkylglycerol monooxygenase [EC:1.14.16.5] | 100% | [alkylglycerol monooxygenase [*Tribolium castaneum*]](https://blast.ncbi.nlm.nih.gov/Blast.cgi) |
| 14 | comp24707_c0_seq1.1.365.minus.CS_1 | 26-hydroxylase [EC:1.14.-.-] | 100% | [cytochrome P450 monooxygenase CYP18A1 [*T. molitor*]](https://blast.ncbi.nlm.nih.gov/Blast.cgi) |
| 15 | comp15334_c0_seq1.1.1094.minus.CK_1 | Lipase [EC 3.1.1.3] | 79.64% | lipase-related protein 1 isoform X2 [*T. castaneum*] |
| 16 | comp8915_c0_seq1.1.441.minus.CS_1 | Esterase [EC 3.1.1.-] | 91.48% | [pancreatic triacylglycerol lipase [*T. castaneum*]](https://blast.ncbi.nlm.nih.gov/Blast.cgi) |
| 17 | comp17156_c0_seq1.1.399.minus.CS_1 | Aldehyde dehydrogenase family [EC:1.2.1.3] | 58.47% | aldehyde dehydrogenase [*T. castaneum*] |
| 18 | comp8911_c0_seq1.1.305.minus.PS_1 | $\alpha/\beta$hydrolase_1, Abhydrolase_6 [EC 3.1.-.-] | 60.40% | [gastric triacylglycerol lipase [*Tribolium castaneum*]](https://blast.ncbi.nlm.nih.gov/Blast.cgi) |
| 19 | comp16428_c0_seq1.1.933.minus.CS_1 | Fatty acid hydroxylase superfamily [EC:1.14.-.-] | 83.55% | [alkylglycerol monooxygenase [*Tribolium castaneum*]](https://blast.ncbi.nlm.nih.gov/Blast.cgi) |
| 20 | comp13777_c0_seq1.43.1113.plus.PS_1 | Alcohol dehydrogenase [EC:3.1.2.12] | 87.29% | [sorbitol dehydrogenase [*Tribolium castaneum*]](https://blast.ncbi.nlm.nih.gov/Blast.cgi) |
| 21 | comp10960_c0_seq1.1.453.minus.CK_1 | $\alpha/\beta$hydrolase family [EC 3.1.1.-] | 83.33% | [protein phosphatase methylesterase 1 [*Asbolus* *verrucosus*]](https://blast.ncbi.nlm.nih.gov/Blast.cgi) |
| 22 | comp10814_c0_seq2.52.786.plus.CK_1 | Redoxin [EC:1.11.1.9] | 77.82%% | [glutathione peroxidase [*Enterobacterales*]](https://blast.ncbi.nlm.nih.gov/Blast.cgi) |
| 23 | comp12658_c0_seq1.1.371.minus.PS_1 | Cu-oxidase [EC 1.10.3.-] | 83.74% | [laccase 1 [*Tribolium castaneum*]](https://blast.ncbi.nlm.nih.gov/Blast.cgi) |
| 24 | comp7426_c0_seq1.1.440.minus.CK_1 | $\alpha/\beta$ hydro_lipase [EC 3.1.-.-] | 74.13% | [lipase 3 [*Tribolium castaneum*]](https://blast.ncbi.nlm.nih.gov/Blast.cgi) |
| 25 | comp4515_c0_seq1.1.419.minus.CS | Cytochrome P450 family 6 [EC:1.14.-.-] | 48.20% | Cytochrome P450 6l1 [*Blattella germanica*] |
| 26 | comp11828_c0_seq1.1.348.minus.CS | Cytochrome P450 family 4 [EC:1.14.-.-] | 42.06% | [Cytochrome P450 4C1 [*Blaberus discoidalis*]](https://blast.ncbi.nlm.nih.gov/Blast.cgi#alnHdr_P29981) |
| 27 | comp16162_c0_seq1.1.802.minus.CK | Laccase-like multicopper oxidase [EC 1.10.3.-] | 23.78% | [Laccase-3](https://blast.ncbi.nlm.nih.gov/Blast.cgi#alnHdr_Q02079) |
| 28 | Feces_k97_48640_gene_4_1 | Laccase-like multicopper oxidase CueO [EC 1.10.3.-] | 79% | Multicopper oxidase CueO [*Escherichia coli K-12*] |
| 29 | comp17350_c0_seq1.15.806.minus.CS | Aldehyde dehydrogenase family [EC:1.2.1.3] | 60.54% | Aldehyde dehydrogenase 1A1 [*Gallus gallus*] |
| 30 | comp5385_c0_seq1.1.548.minus.PS | Alcohol dehydrogenase GroES-like domain [EC:3.1.2.12] | 57.22% | alcohol dehydrogenase [*Homo sapiens*] |
| 31 | comp12941_c0_seq1.1.766.minus.PS | Long-chain-fatty-acid--CoA ligase [EC:6.2.1.3] | 49.59% | Long-chain-fatty-acid--CoA ligase ACSBG2 [*Xenopus laevis*] |
| 32 | comp14315_c1_seq1.1.1066.minus.CK | Acetyl xylan esterase [EC 3.1.1.72] | 48.70% | Chitin deacetylase 8 [*Bombyx mori*] |
| 33 | comp12231_c0_seq1.1.455.minus.CS | Acyl-CoA oxidase [EC:1.3.3.6] | 37.60% | Peroxisomal acyl-coenzyme A oxidase 1 [*Rattus norvegicus*] |
| 34 | comp17495_c0_seq1.1.744.minus.CS | Carboxyl esterase [EC 3.1.1.1] | 42.92% | Carboxylesterase-6 [*Apis mellifera*] |
| 35 | comp12927_c0_seq1.1.611.minus.CS | Endo-beta-1,3(4)-glucanase [EC 3.2.1.6] | 33.33% | Endoglucanase 10 [*Arabidopsis thaliana*] |
| 36 | comp13789_c0_seq1.1.1217.minus.PS | Feruloyl esterase [EC 3.1.1.73] | 53.33% | dipeptidyl peptidase 4 [*Apis mellifera*] |

**Table S9.** Hydrolytic enzymes and relative abundance of enzyme sources in PS, CS and CB-fed mealworm.

|  | **Gene ID** | **KEEG/CAZy annotation** | **Enzyme sources** | **Abundance of Meta-transcriptomic** | | | **Abondance of 16S rRNA microbes** | | | |
| --- | --- | --- | --- | --- | --- | --- | --- | --- | --- | --- |
|  |  |  |  | **CB** | **PS** | **CS** | **CB** | **PS** | | **CS** |
| 1 | comp10814_c0_seq2.52.786.plus.CK_1 | Redoxin [EC:1.11.1.9] | *[Hafniaceae [](https://blast.ncbi.nlm.nih.gov/Blast.cgi)**[Enterobacteriaceae]](https://blast.ncbi.nlm.nih.gov/Blast.cgi)* | 0.34746 | 0.00087 | 0.00111 | 0.021 | | 0.053 | 0.018 |
| 2 | Feces_k97_48640_gene_4_1 | Laccase-like multicopper oxidase CueO [EC 1.10.3.-] | *Kluyvera* | 0.00516 | 0.00941 | 0.01456 | 0.17 | | 0.043 | 0.18 |
| 3 | comp8347_c0_seq1.1.1398.minus.CK_1 | Catalase [EC:1.11.1.6] | *Hafnia [enterobacteria]* | 0.99798 | 0.00174 | 0.00121 | 0.18 | | 0.041 | 0.015 |
| 4 | comp26924_c0_seq1.1.288.minus.CK_1 | Catalase-peroxidase [EC:1.11.1.21] | *Kluyvera* | 0.00516 | 0.00941 | 0.01456 | 0.17 | | 0.043 | 0.18 |
| 5 | comp14052_c0_seq2.1.304.minus.CK_1 | Alkyl hydroperoxide reductase subunit F [EC:1.6.4.-] | *Citrobacter [Enterobacteriacea]* | 0.05566 | 0.00742 | 0.00454 | 0.021 | | 0.053 | 0.018 |
| 6 | comp34094_c0_seq1.52.356.minus.PS_1 | Superoxide dismutase, Fe-Mn family [EC:1.15.1.1] | *[Bacillus](https://blast.ncbi.nlm.nih.gov/Blast.cgi)* | 0.01888 | 0.01390 | 0.00792 | 9.7*10^-5^ | | 8.7*10^-5^ | 12.0*10^-5^ |
| 7 | comp22182_c0_seq1.67.551.minus.CK_1 | Putative oxidoreductase | *Hafnia [enterobacteria]* | 0.99798 | 0.00174 | 0.00122 | 0.18 | | 0.041 | 0.015 |
| 8 | comp21565_c0_seq1.1.367.minus.CK_1 | D-lactate dehydrogenase [EC:1.1.1.28] | *Hafnia* [*Serratia*] | 0.99798 | 0.00174 | 0.00122 | 5.2*10^-5^ | | 2.5*10^-4^ | 1.0*10^-4^ |
| **Sources from host enzymes** | | | | | | | | | | |
| 9 | comp5385_c0_seq1.1.548.minus.PS | Alcohol dehydrogenase GroES-like domain [EC:3.1.2.12] | *Tenebrio molitor* | 14.9220 | 18.5205 | 13.9281 |  | |  |  |
| 10 | comp11737_c0_seq1.1.365.minus.CS_1 | Peroxidase [EC:1.11.1.7] | [*Tribolium castaneum*](https://blast.ncbi.nlm.nih.gov/Blast.cgi) | 68.2946 | 71.5613 | 72.4933 |  | |  |  |
| 11 | comp7761_c0_seq1.1.339.minus.CS_1 | Pyruvate carboxylase [EC:6.4.1.1] | [*Tribolium castaneum*](https://blast.ncbi.nlm.nih.gov/Blast.cgi) | 68.2946 | 71.5613 | 72.4933 |  | |  |  |
| 12 | comp22669_c0_seq1.1.360.minus.CK_1 | Acyloxyacyl hydrolase [EC:3.1.1.77] | [*Tribolium castaneum*](https://blast.ncbi.nlm.nih.gov/Blast.cgi) | 68.2946 | 71.5613 | 72.4933 |  | |  |  |
| 13 | comp16428_c0_seq1.1.933.minus.CS_1 | Alkylglycerol monooxygenase [EC:1.14.16.5] | [*Tribolium castaneum*](https://blast.ncbi.nlm.nih.gov/Blast.cgi) | 68.2946 | 71.5613 | 72.4933 |  | |  |  |
| 14 | comp24707_c0_seq1.1.365.minus.CS_1 | 26-hydroxylase [EC:1.14.-.-] | *Tenebrio molitor* | 14.9220 | 18.5205 | 13.9281 |  | |  |  |
| 15 | comp15334_c0_seq1.1.1094.minus.CK_1 | Lipase [EC 3.1.1.3] | [*Tribolium castaneum*](https://blast.ncbi.nlm.nih.gov/Blast.cgi) | 68.2946 | 71.5613 | 72.4933 |  | |  |  |
| 16 | comp8915_c0_seq1.1.441.minus.CS_1 | Esterase [EC 3.1.1.-] | [*Tribolium castaneum*](https://blast.ncbi.nlm.nih.gov/Blast.cgi) | 68.2946 | 71.5613 | 72.4933 |  | |  |  |
| 17 | comp17156_c0_seq1.1.399.minus.CS_1 | Aldehyde dehydrogenase family [EC:1.2.1.3] | [*Tribolium castaneum*](https://blast.ncbi.nlm.nih.gov/Blast.cgi) | 68.2946 | 71.5613 | 72.4933 |  | |  |  |
| 18 | comp8911_c0_seq1.1.305.minus.PS_1 | $\alpha/\beta$hydrolase_1, Abhydrolase_6 [EC 3.1.-.-] | [*Tribolium castaneum*](https://blast.ncbi.nlm.nih.gov/Blast.cgi) | 68.2946 | 71.5613 | 72.4933 |  | |  |  |
| 19 | comp13777_c0_seq1.43.1113.plus.PS_1 | Alcohol dehydrogenase [EC:3.1.2.12] | [*Tribolium castaneum*](https://blast.ncbi.nlm.nih.gov/Blast.cgi) | 68.2946 | 71.5613 | 72.4933 |  | |  |  |
| 20 | comp10960_c0_seq1.1.453.minus.CK_1 | $\alpha/\beta$hydrolase family [EC 3.1.1.-] | [*Tribolium castaneum*](https://blast.ncbi.nlm.nih.gov/Blast.cgi) | 68.2946 | 71.5613 | 72.4933 |  | |  |  |
| 21 | comp12658_c0_seq1.1.371.minus.PS_1 | Cu-oxidase [EC 1.10.3.-] | [*Tribolium castaneum*](https://blast.ncbi.nlm.nih.gov/Blast.cgi) | 68.2946 | 71.5613 | 72.4933 |  | |  |  |
| 22 | comp7426_c0_seq1.1.440.minus.CK_1 | $\alpha/\beta$ hydro_lipase [EC 3.1.-.-] | [*Tribolium castaneum*](https://blast.ncbi.nlm.nih.gov/Blast.cgi) | 68.2946 | 71.5613 | 72.4933 |  | |  |  |
| 23 | comp4515_c0_seq1.1.419.minus.CS | Cytochrome P450 family 6 [EC:1.14.-.-] | *Tenebrio molitor* | 14.9220 | 18.5205 | 13.9281 |  | |  |  |
| 24 | comp11828_c0_seq1.1.348.minus.CS | Cytochrome P450 family 4 [EC:1.14.-.-] | *Tenebrio molitor* | 14.9220 | 18.5205 | 13.9281 |  | |  |  |
| 25 | comp16162_c0_seq1.1.802.minus.CK | Laccase-like multicopper oxidase [EC 1.10.3.-] | [*Tribolium castaneum*](https://blast.ncbi.nlm.nih.gov/Blast.cgi) | 68.2946 | 71.56137 | 72.4933 |  | |  |  |
| 26 | comp17350_c0_seq1.15.806.minus.CS | Aldehyde dehydrogenase family [EC:1.2.1.3] | [*Tribolium castaneum*](https://blast.ncbi.nlm.nih.gov/Blast.cgi) | 68.2946 | 71.5613 | 72.4933 |  | |  |  |
| 27 | comp12941_c0_seq1.1.766.minus.PS | Long-chain-fatty-acid--CoA ligase [EC:6.2.1.3] | [*Tribolium castaneum*](https://blast.ncbi.nlm.nih.gov/Blast.cgi) | 68.2946 | 71.5613 | 72.4933 |  | |  |  |
| 28 | comp14315_c1_seq1.1.1066.minus.CK | Acetyl xylan esterase [EC 3.1.1.72] | [*Tribolium castaneum*](https://blast.ncbi.nlm.nih.gov/Blast.cgi) | 68.2946 | 71.5613 | 72.4933 |  | |  |  |
| 29 | comp12231_c0_seq1.1.455.minus.CS | Acyl-CoA oxidase [EC:1.3.3.6] | [*Tribolium castaneum*](https://blast.ncbi.nlm.nih.gov/Blast.cgi) | 68.2946 | 71.5613 | 72.4933 |  | |  |  |
| 30 | comp17495_c0_seq1.1.744.minus.CS | Carboxyl esterase [EC 3.1.1.1] | [*Tenebrio molitor*](https://blast.ncbi.nlm.nih.gov/Blast.cgi#alnHdr_AKZ17680) | 14.9220 | 18.5205 | 13.9281 |  | |  |  |
| 31 | comp12927_c0_seq1.1.611.minus.CS | Endo-beta-1,3(4)-glucanase [EC 3.2.1.6] | [*Tribolium castaneum*](https://blast.ncbi.nlm.nih.gov/Blast.cgi) | 68.2946 | 71.5613 | 72.4933 |  | |  |  |
| 32 | comp13789_c0_seq1.1.1217.minus.PS | Feruloyl esterase [EC 3.1.1.73] | [*Tribolium castaneum*](https://blast.ncbi.nlm.nih.gov/Blast.cgi) | 68.2946 | 71.5613 | 72.4933 |  | |  |  |

Notes: [] represented 16S rRNA bacteria name.


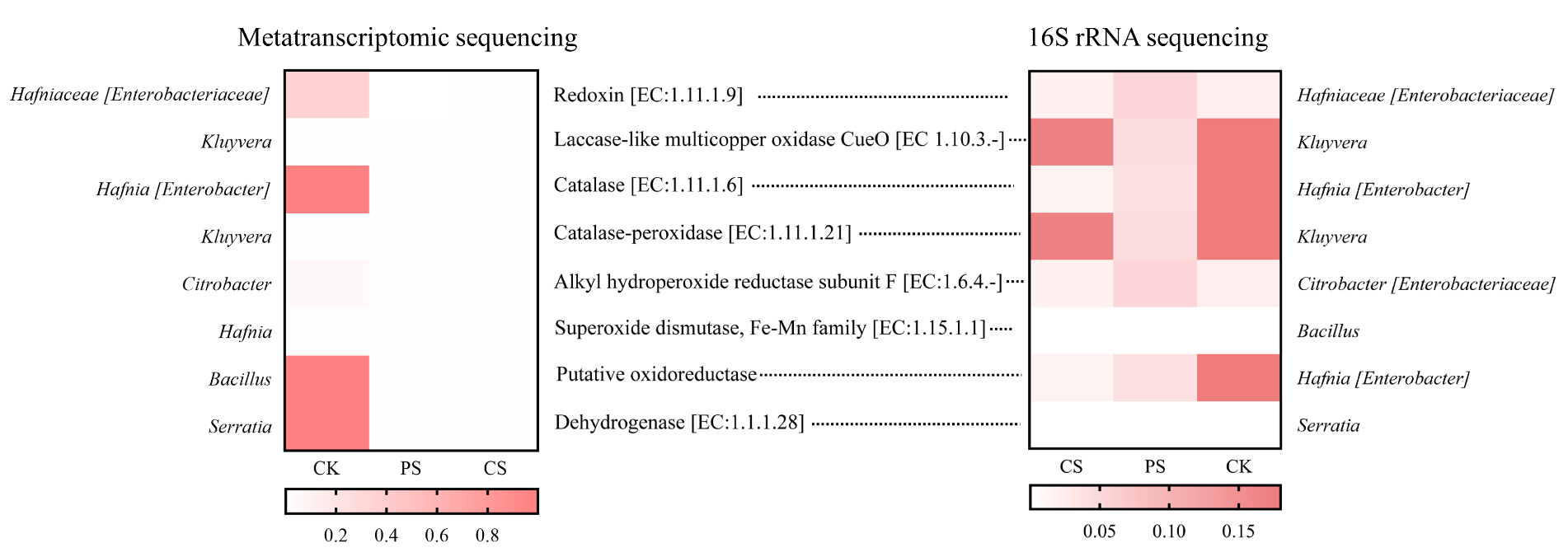


**Fig. S10** Relative abundance of bacterial species in the 16S rRNA bacterial community and meta-transcriptomic taxonomy analysis associated with the upregulated genes for PS/CS biodegradation.


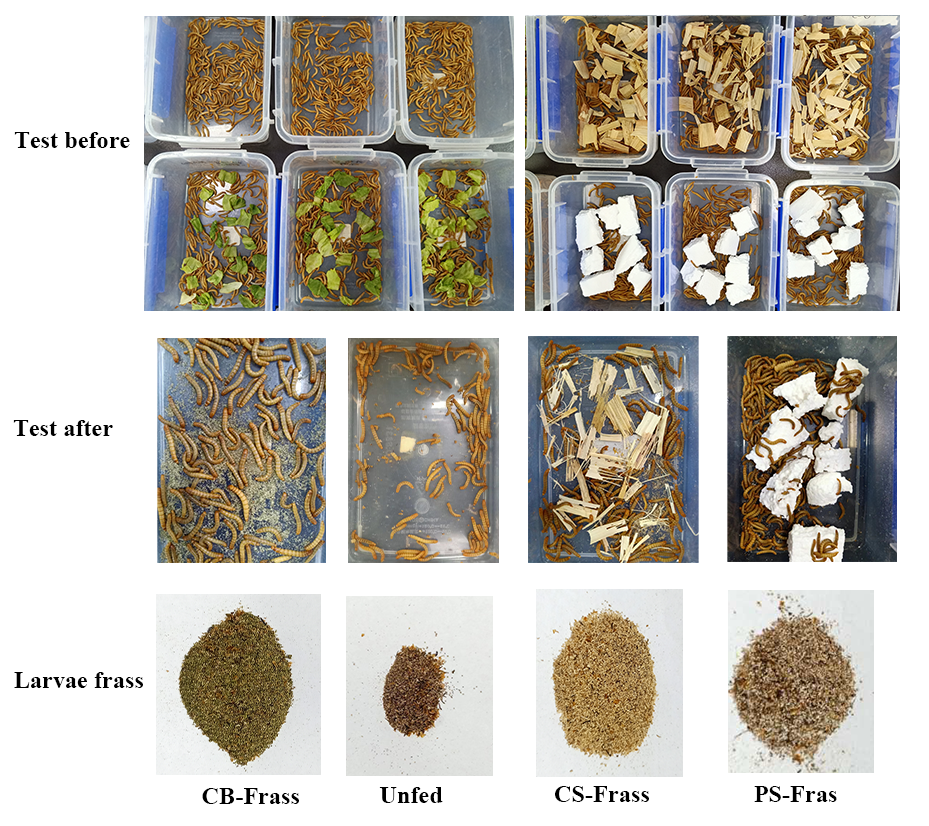


**Fig. S1** Images of the mealworms before and after feeding different (CB, PS, CS) feedstocks and unfed.


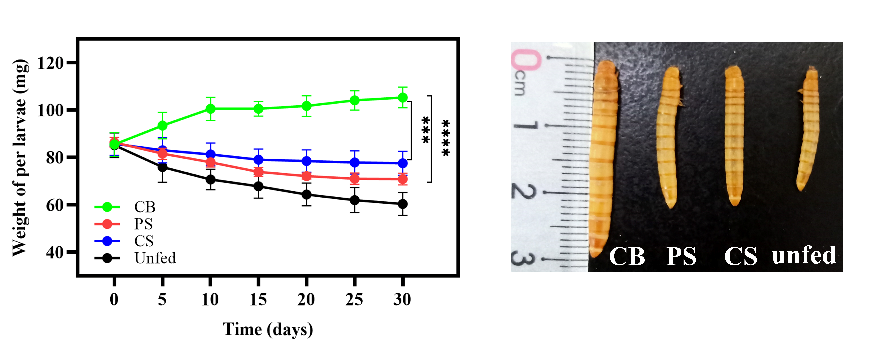


**Fig. S2** The average weight of mealworms fed with different feedstocks in the 30-days test.


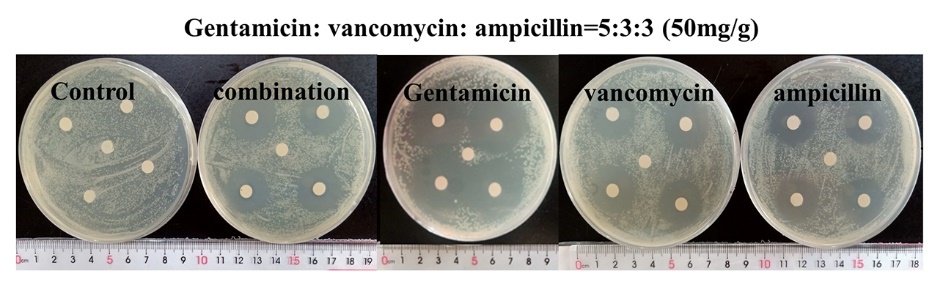


**Fig. S3** Inhibition halos of the growth of gut microbiota from mealworms by the antibiotics (6 mg antibiotic per disk, weight ratio, gentamicin: vancomycin: ampicillin = 5:3:3). The disks of control plate and the central disk in antibiotic plate were inoculated with sterile water and didn’t form inhibition halo.


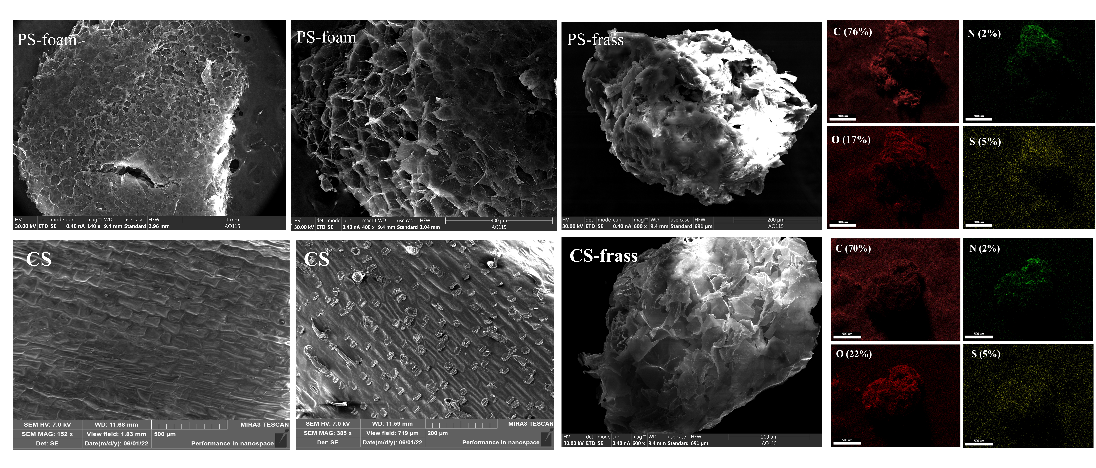


**Fig. S4** The SEM and EDS micrographs of CS, PS foam and their excreted feces by larvae fed with PS and CS.


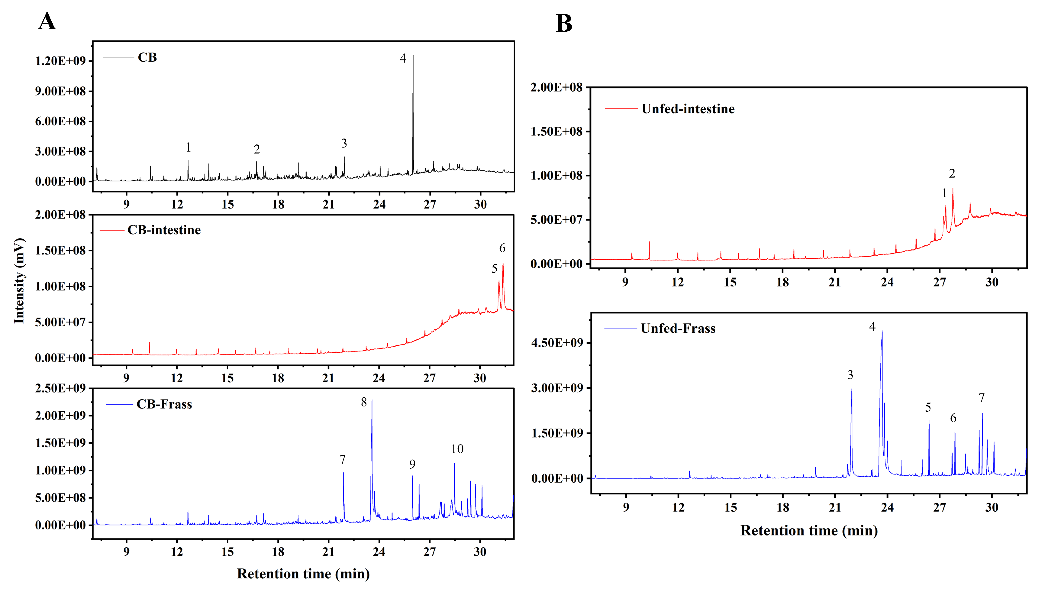


**Fig. S5** GC-MS analysis of intestinal and frass samples from CB-fed and unfed mealworms. Compounds in figure (a): 1. Azulene (C_10_H_8_), 2. Hexadecane (C_16_H_34_), 3. 21.92:1,2-Benzenedicarboxylic acid (C_16_H_22_O_4_), 4. Retinoic acid, methyl ester(C_21_H_30_O_2_), 5. Vitamin A aldehyde (C_20_H_28_O), 6. 1,2,3,4,5,6-Hexahydro-1,1,5 ,5-tetramethyl-2,4a-methan onaphthalen-7(4aH)-one (C_15_H_22_O), 7. Pentadecanoic acid (C_15_H_30_O_2_), 8. Ricinoleic acid (C_18_H_34_O_3_), 9. 2,5-Cyclohexadiene-1,4-dione (C_14_H_20_O_2_), 10. Tetradecanoate (C_41_H_72_O_2_); (b): 1-2, Prednisolone Acetate (C_23_H_30_O_6_), 3. Pentadecanoic acid (C_15_H_30_O_2_), 4. 9-Octadecenoic acid (Z)-, methyl ester (C_19_H_36_O_2_), 5. Eicosane (C_20_H_42_), 6-7. Tetracosane (C_24_H_50_).


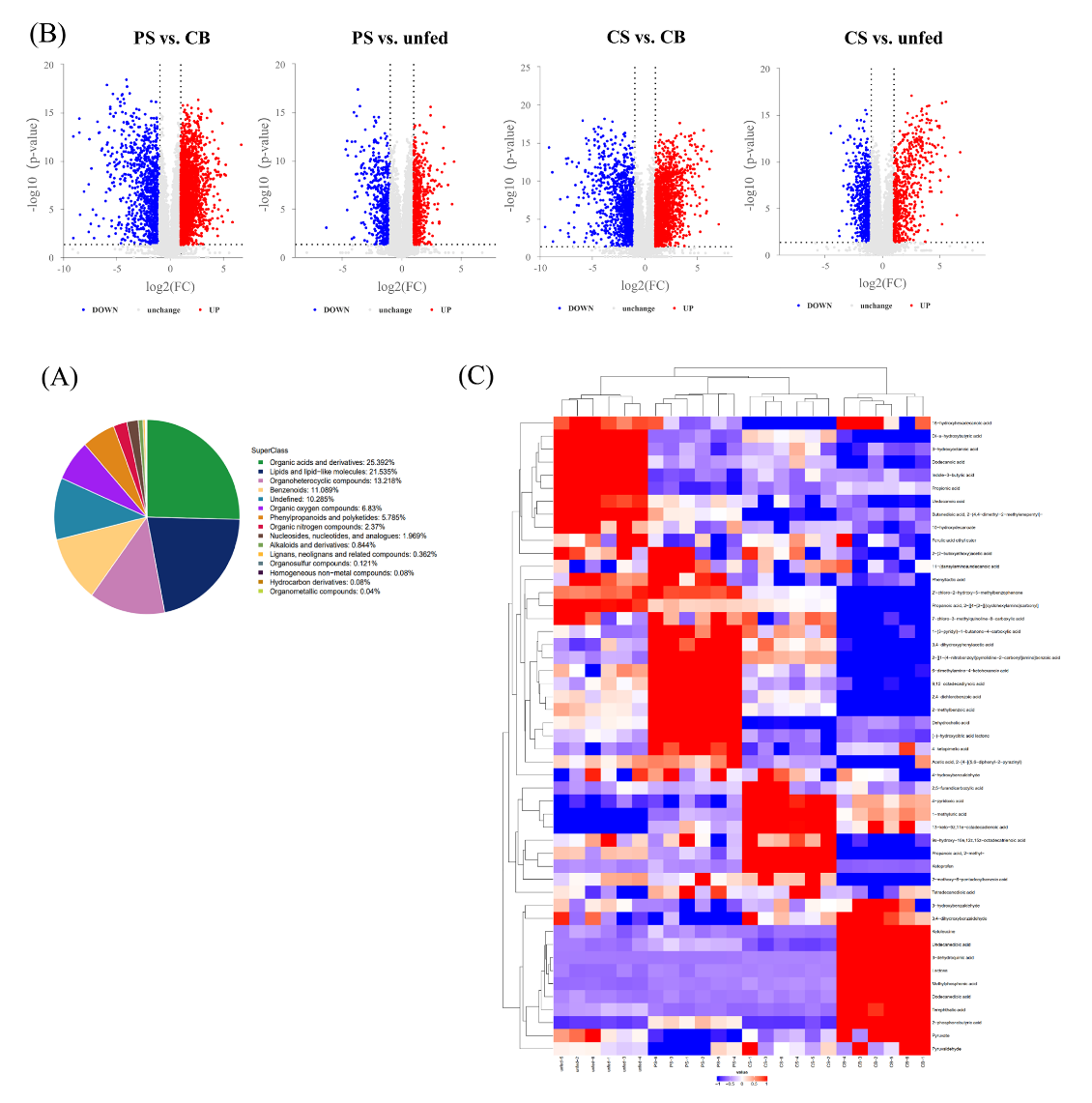


**Fig. S6** The comparative metabolomic analysis of mealworms fed with different feedstocks. (A) The number proportion of identified metabolites in each chemical classification. (B) Volcanic map of differential metabolites. (C) Positive ion pattern significant difference metabolite hierarchy clustering heat map.


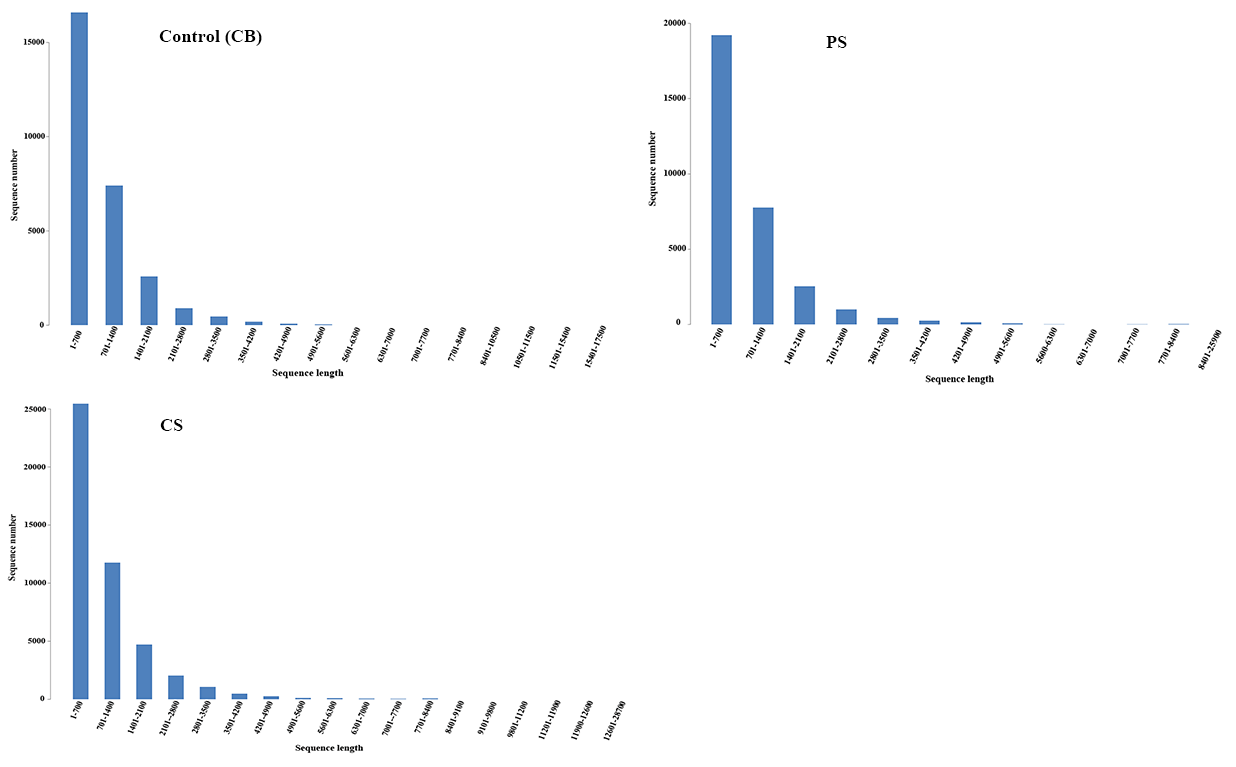


**Fig. S7** The sequence length distribution of four groups.


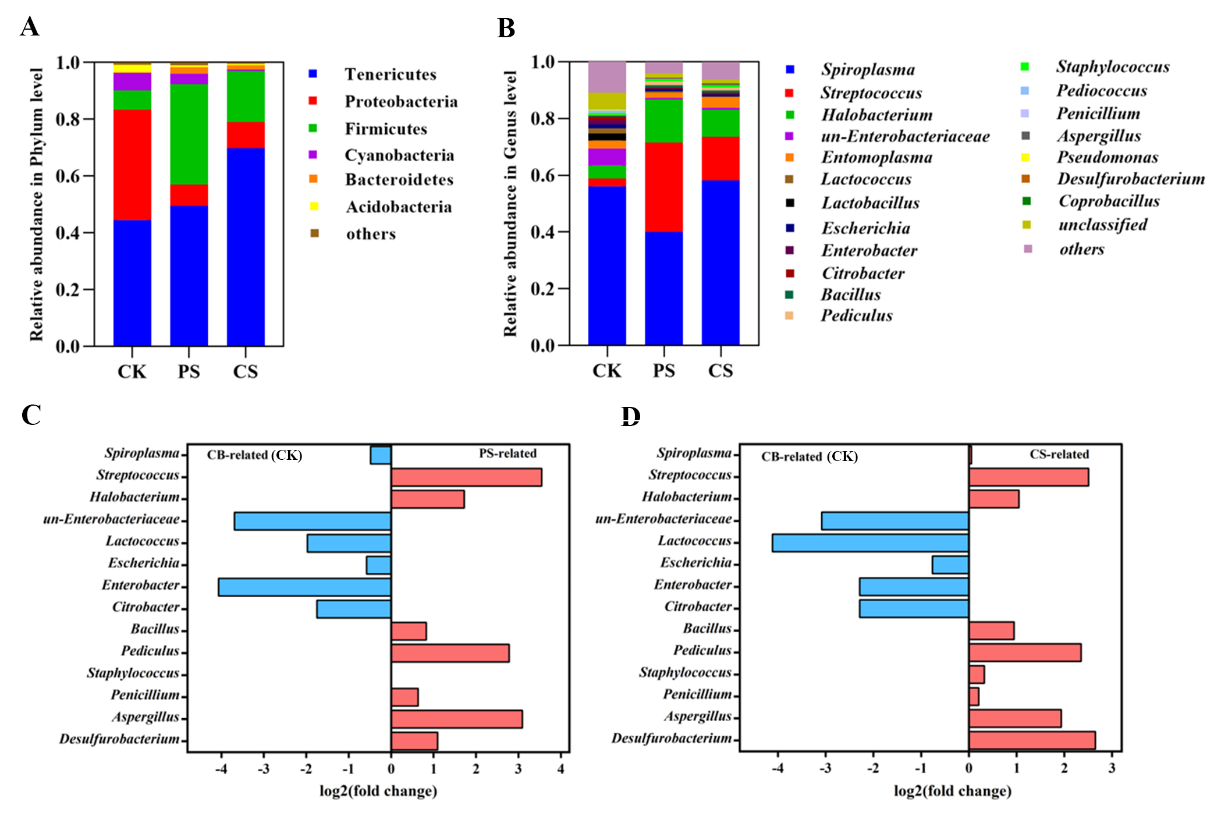


**Fig. S8** Relative abundance of the active gut microbiomes fed with different diets at the phylum (A) and the genus levels (B). Differential abundance analysis of between CB-fed vs.PS-fed (C), CB-fed vs. CS-fed (D). Genus only presented in one of the two diets are expressed with log2 fold change.

**
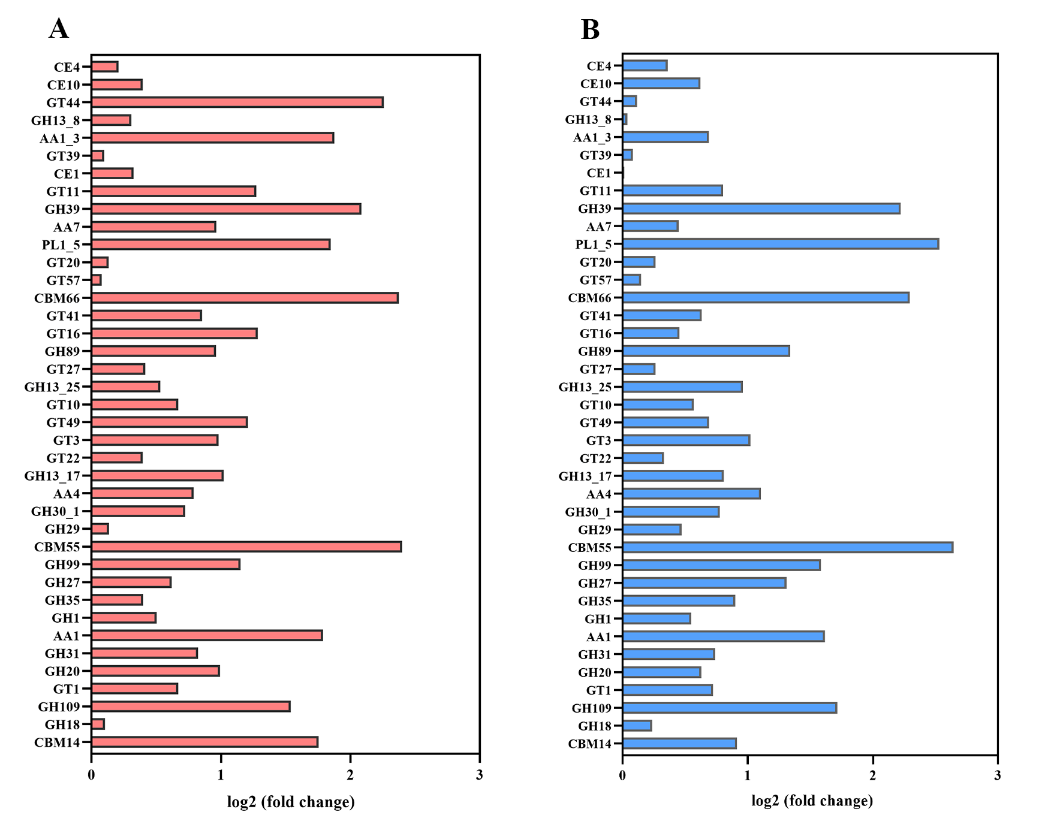
**

**Fig. S9** The CAZymes family protein relative abundance in the PS-fed (A) and CS-fed larvae (B).


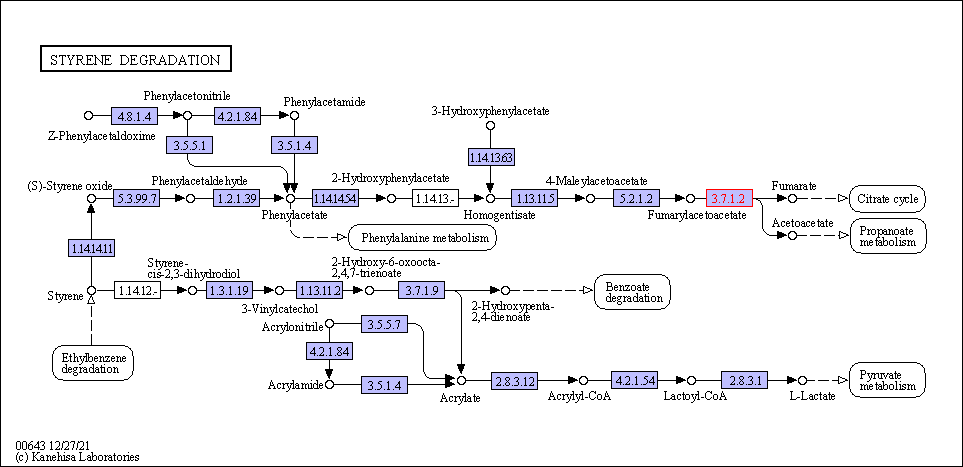


**Fig. S11** The monomer styrene of [polystyrene](https://www.sciencedirect.com/topics/earth-and-planetary-sciences/polystyrene) (PS) degradation pathway in the gut of *T. molitor* larva based on comparative metabolic analysis.


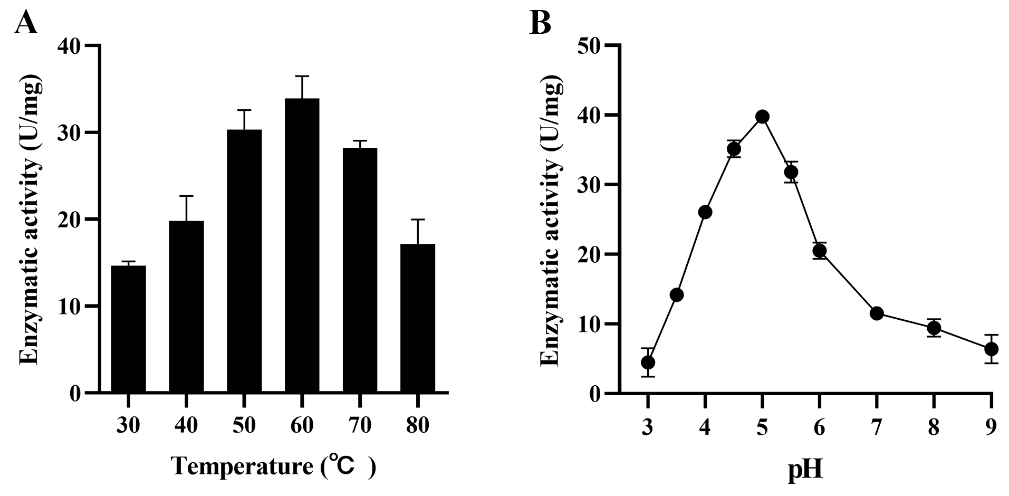


**Fig. S12** The laccase activity of Lac640. (A) Effect of temperature on Lac640 activity. (B) Effect of pH on Lac640 activity.
